# Improved Selectivity and Stability in Methane Dry Reforming by Atomic Layer Deposition on Ni-CeO_2_–ZrO_2_/Al_2_O_3_ Catalysts

**DOI:** 10.1021/acscatal.4c02019

**Published:** 2024-05-30

**Authors:** Jonathan Lucas, Nirenjan Shenoy Padmanabha Naveen, Michael J. Janik, Konstantinos Alexopoulos, Gina Noh, Divakar Aireddy, Kunlun Ding, James A. Dorman, Kerry M. Dooley

**Affiliations:** † Department of Chemical Engineering, Louisiana State University, Baton Rouge, Louisiana 70803, United States; ‡ Department of Chemical Engineering, The Pennsylvania State University, University Park, Pennsylvania 16802, United States

**Keywords:** dry reforming, CeO_2_/Al_2_O_3_, atomic layer deposition, DFT, XAFS characterization

## Abstract

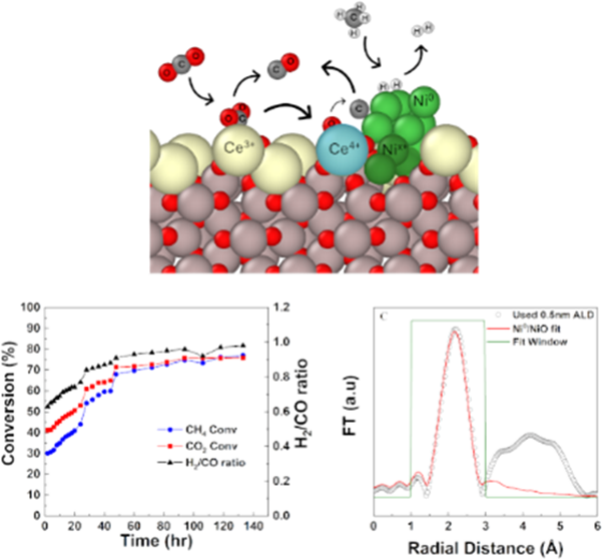

Ni can be used as a catalyst for dry reforming of methane (DRM), replacing more expensive and less abundant noble metal catalysts (Pt, Pd, and Rh) with little sacrifice in activity. Ni catalysts deactivate quickly under realistic DRM conditions. Rare earth oxides such as CeO_2_, or as CeO_2_–ZrO_2_–Al_2_O_3_ (CZA), are supports that improve both the activity and stability of Ni DRM systems due to their redox activity. However, redox-active supports can also enhance the undesired reverse water gas shift (RWGS) reaction, reducing the hydrogen selectivity. In this work, Ni on CZA was coated with an ultrathin Al_2_O_3_ overlayer using atomic layer deposition (ALD) to study the effects of the overlayer on catalyst activity, stability, and H_2_/CO ratio. A low-conversion screening method revealed improved DRM activity and lower coking rate upon the addition of the Al_2_O_3_ ALD overcoat, and improvements were subsequently confirmed in a high-conversion reactor at long times onstream. The overcoated samples gave an H_2_/CO ratio of ∼1 at high conversion, much greater than uncoated catalysts, and no evidence of deactivation. Characterization of used (but still active) catalysts using several techniques suggests that active Ni is in formal oxidation state >0, Ni–Ce–Al is most likely present as a mixed oxide at the surface, and a nominal thickness of 0.5 nm for the Al_2_O_3_ overcoat is optimal.

## 1 Introduction

Chemical transformation of CO_2_ could reduce greenhouse gas emissions and lead to a more carbon-neutral petrochemical industry.^1,2^ Due to the high stability of the CO_2_ molecule, chemical transformation is usually achieved through catalytic processes such as dry reforming of methane (DRM), hydrogenation (Sabatier reaction), formation of alkyl carbonates, etc.^3−5^ In particular, DRM has the potential to transform the two major greenhouse gases, CH_4_ and CO_2_, into syngas (H_2_, CO) at close to a 1:1 ratio, a mixture that would still require a water–gas shift to a higher ratio to render it usable for downstream processes such as Fischer–Tropsch.^6^

Ni-based DRM catalysts have received special attention over the past few decades due to their economic advantage over noble metal catalysts (Pt, Pd, Rh) and promising catalytic performance.^7−9^ However, at the characteristically harsh DRM conditions (*T* > 700 °C, P of 1–10 bar) Ni is highly susceptible to deactivation by both coking and sintering. Sintering and loss of the active surface area are significant issues in Ni-based DRM.^10−12^ The sintering of the active Ni metal can eventually lead to uncontrollable coke formation, by either the Boudouard (2CO ↔ C(s) + CO_2_) or methane decomposition (CH_4_ → 2H_2_ + C(s)) reactions.^13−16^

Cerium oxide has attracted considerable attention as a reducible support in high-temperature reactions due to the facile interchange between Ce^3+^/Ce^4+^ oxidation states.^17−19^ This semireducible aspect of ceria means oxygen vacancies (OVs) are formed. The vacancies are sites for the adsorption of CO_2_, which is reduced to CO, eliminating two vacancies.^20−22^ Oxide–CeO_2_ mixtures can further optimize the oxygen storage capacity (OSC) associated with such redox behavior. Doping the CeO_2_ lattice with Zr increases the OSC because the Ce–O bond is weakened when the fluorite crystal structure is distorted by intercalation of the smaller Zr^4+^ ion.^23−25^ For Ce–Zr–Al mixed oxides (CZAs), 3/1 to 1/1 (molar) Ce/Zr ratios give the largest OSCs and therefore the most potential OV formation.^26−28^ When high concentrations of Zr are present, the fluorite structure converts to a tetragonal phase, reducing the OSC of the support.^29^ Due to high oxygen mobility in certain CZA mixtures, lattice oxygen can readily oxidize surface-deposited carbon.^30^ However, if the rate at which the carbon is oxidized is too slow, or the Ni clusters too large (causing oxygen diffusion through the nickel to take longer), carbon species can intercalate the cluster and form filaments.^14,31^ The activation of CO_2_ by surface OVs also lowers the observed activation energy of DRM.^32−35^ CO_2_ can adsorb and get reduced by Ce^3+^ defect sites even without a nearby Ni active center, or a hydroxyl-terminated surface.^33^ However, while the OVs present in CeO_2_ and CZA improve DRM activity, they also enhance the activity of the RWGS reaction, thus lowering the H_2_/CO ratio.^22,36^

Using a shell layer to confine Ni during DRM could inhibit sintering at elevated temperatures, especially >700 °C. Atomic layer deposition (ALD) is a controllable self-limiting surface reaction method for the deposition of oxide layers. Traditionally, ALD processes are described as generating conformal layers stemming from the self-limiting nature of the technique.^37−41^ Recently, this description has evolved to account for disparate incubation times for film nucleation on oxide and metal surfaces, based on the ability to form hydroxylated surfaces as predicted by thermodynamics.^42−44^ Understanding the selective deposition process allows for the growth of thin layers on oxide surfaces without covering the catalytic metal sites. ALD overcoats have the potential to act as aggregation barriers, and as electronic modifiers of transition metals (TMs) to increase selectivity.^45−48^ Furthermore, Al_2_O_3_ is a simple nonreducible oxide to deposit via ALD, and has been shown to limit hydrogen spillover and diffusion compared to more reducible oxides such as TiO_2_.^49^ The efficacy of thin shell layers, such as those produced by ALD or other core–shell techniques, is open to dispute, at least for DRM. While some core–shell catalysts (with Ni initially deposited on the core) have shown impressive turnover frequencies, such as a Ni/CeO_2_@SiO_2_ catalyst (turnover frequency ∼0.39 s^–1^ on a total Ni atom basis),^50^ it is not known if these are truly layered materials, or if oxide and/or Ni mixing into the shell is taking place. Research on ALD for Ni-based DRM catalysts has mainly focused on coated Ni/Al_2_O_3_ catalysts to prevent aggregation of Ni particles.^51−53^ There is debate about using alumina ALD overlayers due to possible formation of NiAl_2_O_4_, which is inactive for DRM.^51,54^ But Al_2_O_3_ ALD overlayers have decreased Ni particle aggregation during DRM,^51,52,55^ even though thick Al_2_O_3_-overcoated Ni–Al_2_O_3_ catalysts still deactivated rapidly after reaching maximum activity, and selectivity (H_2_/CO ratio) was not explored.^56^ More recently, there have been conflicting reports on alumina-coated SiO_2_ and CeO_2_, concluding that both thick (4 nm)^57^ and thin (1 ALD cycle)^58^ result in excellent catalytic performance, though long-term coking effects were not explored. This raises the question about the role of the overlayer with the support, metal, and adsorbate species. We hypothesize that ultrathin nonreducible Al_2_O_3_ layers can enhance catalyst performance by limiting both Ni aggregation and adsorbate spillover due to selective deposition on the ceria surface.

In this work, we demonstrate how a nonreducible overlayer can drastically improve the selectivity and stability of a Ni-CZA catalyst. The addition of a 0.5 nm ALD Al_2_O_3_ overlayer to a Ni-CZA core increased the lifetime of the uncoated catalyst and the measured coking rate by at least 10-fold. The Ni clusters of the catalyst used were also smaller and less reducible. This structure had the added benefit of decreasing, in absolute terms, the RWGS rate and increasing the DRM rate.

## 2 Methods

### 2.1 Materials

Commercial CZA40 support (Al_2_O_3_ 40.07 wt %, ZrO_2_ 23.59 wt %, CeO_2_ 33.47 wt %, La_2_O_3_ 1.45 wt %, Y_2_O_3_ 1.42 wt %, Lot #CZLYA40-150802-AJ) was supplied by PIDC. Other chemicals such as nickel nitrate hexahydrate (Ni(NO_3_)_2_·6H_2_O, Sigma-Aldrich, 99%), trimethylaluminum (Al(CH_4_)_3_, Sigma-Aldrich, 97%), titanium(IV) isopropoxide (Ti[OCH(CH_3_)_2_]_4_, Sigma-Aldrich, 99%), and urea (CH_4_N_2_O, VWR, ACS grade) were used as received. The reactant gases were CH_4_ (Airgas, 99%) and CO_2_ (Airgas, 99%). Air (Airgas, breathing grade), N_2_ (Airgas, UHP), and 5% H_2_ (Airgas, certified) were also used for calcinations/reductions.

### 2.2 Catalyst Syntheses

Adsorptive deposition (aka “strong electrostatic adsorption”) was used to deposit 4 wt % Ni onto CZA40, as adapted from previous work.^13,59^ Powdered CZA40 was added along with the desired amount of Ni(NO_3_)_2_·6H_2_O and 30 mL of 0.3 M urea per g support. The solution was stirred and reacted for 24 h under reflux at 90 °C. The product powder was washed with deionized (DI) water and dried at 100 °C overnight, then reduced as discussed subsequently.

The Al_2_O_3_-ALD method was adapted from previous work.^60^ The AlO_*x*_ overcoats were deposited using a benchtop viscous-flow ALD reactor (GEMStar-6 XT) at 150 °C, alternating exposure of trimethylaluminum (TMA) and water vapor using N_2_ as both carrier and purge gas, with both TMA and water bubblers at room temperature. Each AlO_*x*_ cycle consisted of 90 s of TMA exposure followed by 90 s of water exposure with 300 s of N_2_ purge between each exposure (90s-300s-90s-300s). Three to 12 cycles of AlO_*x*_ ALD were performed on Ni/CZA40. The mass gain was obtained by before- and after-ALD weighing. Samples are referred to by the layer thickness increase upon ALD, as 0.3, 0.5, or 1.2 nm ALD-CZA40.

After calcination in air at 600 °C for 1 h, and reduction in 4% H_2_/Ar at 500 °C, 10% CO_2_/Ar was pulsed over the catalysts at various temperatures. The conformality of the coatings was examined by the adsorption of CO_2_ (Nicolet Nexus 670 FTIR, DRIFTS mode, MCT/A detector, 4 cm^–1^ resolution, and 10^–6^ Torr).

### 2.3 X-ray Diffraction (XRD)

Powder X-ray diffraction (XRD) was performed to analyze the bulk structure of the as-synthesized and used catalyst samples. Diffractograms were collected using a PANalytical XRD at 45 kV and 40 mA. Spectra were recorded at 0.04° steps over the range of 5–70°, with a dwell time of 60 s. A Cu Kα radiation source was used. The average crystal size of an identified phase was calculated using the Scherrer equation , where *K* is the dimensionless shape factor, typically set at 0.9, λ is the X-ray wavelength for Cu Kα radiation, and β is the full width at half-maximum of the XRD peak analyzed.

### 2.4 Porosimetry

The surface area, total pore volume, and pore size distributions of the fresh and used catalysts were measured by N_2_ adsorption by using a Micromeritics ASAP 2020 Plus porosimeter. Samples were dried at 300 °C and degassed prior to analysis. The surface area was determined by the Brunauer–Emmett–Teller (BET) method and the pore size distribution by the BJH method.

### 2.5 High-Resolution Transmission Electron Microscopy (HRTEM) and Energy-Dispersive X-ray (EDX) Spectroscopy

The morphology and size of the catalysts were analyzed by HRTEM using a 200 kV JEOL NEARM electron microscope at Oak Ridge National Laboratory equipped with double aberration correctors, a dual-energy-loss spectrometer, and a cold FEG source. Before imaging, the samples were dispersed in ethanol and drop cast on a 300 mesh lacey carbon grid. EDX was performed using an FEI Quanta 3D FIB microscope equipped with an EDAX Apollo XL detector operating at an accelerating voltage of 20 kV and a current of 4 nA. Due to the strength of the TEM electron beam at high magnification, it proved impossible to obtain EDX of high magnification images due to sample deformation. Resolution was kept at a minimum of 100 nm. ImageJ (version 1.53k) was used to analyze lattice spacings of imaged catalysts.

### 2.6 XPS and XAS

X-ray photoelectron spectroscopy (XPS) was performed using a Scienta Omicron ESCA 2SR equipped with a monochromatic Al K_α_ (*h*ν = 1486.6 eV) X-ray source and a hemispherical analyzer with a 128-channel detector, at 1.3 × 10^–9^ Torr. The Gaussian width of the photon source was 0.5 eV with a focus voltage of 300 V. The adventitious carbon C 1s peak at 284.4 V was used to calibrate the energies. After Shirley background subtraction, all peaks were fitted by using Casa XPS (version 2.3.25) as Gaussians.

X-ray absorption spectroscopy (XAS) was performed at the Ni K-edge at the LSU Center for Advanced Microstructures and Devices (CAMD). Some spectra were collected at the HEXAS beamline using a Ge 220 double crystal monochromator, at room temperature in fluorescence mode, with a Ni foil calibration standard. Other spectra were collected at the WDCM 2.0 beamline equipped with a Si 111 channel-cut monochromator in fluorescence mode and calibrated with a Ni foil standard. Integration time was adjusted to obtain adequate counts up to a wavenumber of 12. Runs were repeated to improve the counting statistics. Both X-ray absorption near-edge (XANES) and X-ray absorption fine structure (XAFS) spectra were collected.

Background subtraction, deglitching, and merging of spectra were performed using Athena 0.9.061. Ni K-edge XAFS fitting was performed in Artemis 0.9.26. Four parameters were varied to obtain the best possible fits, *S*_0_^2^ (amplitude reduction factor), σ^2^ (Debye–Waller factor), Δ*E*_0_ (deviation in *E*_0_ caused by structural deviations from the ideal crystal structure), and Δ*R* (deviation in interatomic distance). A NiO standard was fitted first to get information about *S*_0_^2^ with known coordination numbers and Δ*R*′s. The fitting range in *R* space was 1–5 Å, and all significant scattering paths were included.

### 2.7 Catalytic Activity Measurement

Catalytic activity was measured at differential conversions using a TA SDT Q600 differential scanning calorimeter (DSC)/Thermogravimetric Analyzer (TGA). ALD-coated catalysts were first pretreated in air (100 mL/min) at 600 °C for 1 h to remove residual water and generate pore space in the overlayer. Then the samples were reduced in 5% H_2_/95% N_2_ for 3 h or as long as necessary at 600 °C. This was sufficient to fully reduce the catalysts prior to the DRM experiments, as shown in Figure S1. We varied the reduction temperature from 500–750 °C in 50 °C increments for Ni-CZA40 and 600 °C reduction gave the highest DRM activity, although over the 550–650 °C range little difference was observed.

DRM (135 mL/min total flow, 0.25 bar partial pressure CH_4_ and CO_2_, 0.5 bar N_2_) took place at 650 and 750 °C for 1.5 h at each temperature. The heat flux and change in mass were both measured. The heat flux is roughly the heat evolved by the DRM reaction, and the weight change can be related to the coking rate.^13^ Heat flow data were used in an Aspen HYSYS program^61^ to calculate the DRM rate and methane conversion. Further details are given in the Supporting Information.

Catalysts showing promising activity at differential conditions were further tested in a fixed-bed reactor, which was a 12.5 mm quartz reactor tube with α-alumina and quartz wool as an extra packing material. Catalysts were reduced for 6 h at 600 °C in flowing 5%H_2_/95% N_2_, with a ramp rate of 10 °C/min. Catalyst weight was varied between 0.35 and 0.25 g to vary GHSV. The feed composition was 1:1 CH_4_/CO_2_ (molar) at ∼0.6 bar of each reactant. The setup here was the same as in previous work.^13^ An Agilent 6890N GC-MS instrument was used to analyze the outlet gas composition. The reactor tube is heated by a furnace (Teco F-5-1000, 320 W) whose temperature is controlled by a Eurotherm 818P PID controller. Figure S2 shows a schematic of the reactor setup. Information about product analysis is also in the Supporting Information (Table S1). Conversion, yield, and selectivity from these experiments were calculated from the extent of reaction. Three equations represent the main reactions (DRM, reverse water–gas shift, and coking):
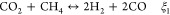
1
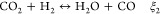
2
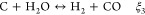
3

The ξ′s are molar extents of reactions in mol/min, calculated by solving the component mass balances simultaneously, using both the compositions and effluent flow rate. These results were refined using a nonlinear regression method where the objective function is the sum of the squared residuals of the CH_4_, CO_2_, H_2_O, and CO and H_2_ mass balances. The terms *F*_in,CH_4__, and *F*_in,CO_2__ are molar flow rates of the feed components in mol/min. The yields of products on an elemental carbon basis and the conversions of CH_4_ and CO_2_, and the turnover frequency (TOF), defined as the amount of CH_4_ reacted per second per total Ni atoms, are calculated as follows:
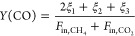
4
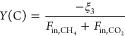
5
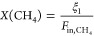
6
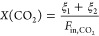
7
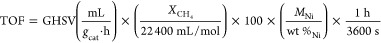
8where *M*_Ni_ is the molecular weight of Ni.

### 2.8 Density Functional Theory (DFT) Calculations

#### 2.8.1 Electronic Structure Methods

DFT calculations were performed using the Vienna *ab initio* Simulation Package (VASP).^62^ The electronic exchange and correlation interactions were described by the generalized gradient approximation (GGA) method with the Perdew–Burke–Ernzerhof (PBE) functional.^63^ The projector augmented wave method was used to represent the core electrons, and a plane wave basis set was used to represent the valence electrons^64^ with an energy cutoff of 500 eV. The Monkhorst–Pack scheme^65^ was used to sample the Brillouin zone, with the third vector perpendicular to the surface. Electronic SCF cycles converged with an energy difference of less than 1 × 10^–5^ eV. Structural optimizations minimized forces on all atoms below 0.05 eV/Å. Spin-polarized calculations were used for systems with unpaired electrons. Slab-to-slab dipole interactions were corrected within each SCF while simulating surfaces. All isolated gas molecules were optimized with a 1 × 1 × 1 *k*-point grid, in a 10 × 10 × 10 Å unit cell.

DFT+*U* corrections were used for structures with Ce, due to the well-established difficulties that DFT faces while representing the 4f orbitals of Ce. A *U* value of 5 was used on the f orbitals of Ce which is consistent with our previous work on ceria-based systems.^13,66,67^ DFT is also known to have difficulties representing the localized d-states of transition metals; thus, *U* corrections were also applied for systems containing Ni. A *U*-value of 6.4 eV^68^ was used on the d orbitals of Ni because of closer agreement between the experimental^69^ and simulated band gap (4.0 vs 3.89 eV).

#### 2.8.2 Bulk and Surface DFT Models

The bulk γ-Al_2_O_3_ structure was obtained from Digne’s model^70^ as it has found extensive applications in DFT-based studies. The two important benefits of using this model for first-principles simulations are the availability of a smaller unit cell and the presence of fully occupied lattice sites, which enable the creation of supercell surface models.^71^ CeAlO_3_ has two stable lowest energy structures. We used the rhombohedral perovskite-type structure, as it is similar to the NdAlO_3_ structure.^72^ The bulk CeO_2_ structure used in this study is the commonly used cubic fluorite-type structure. Geometrically optimized pure CeAlO_3_ bulk and γ-Al_2_O_3_ unit cells are shown in Figure S4.

To simulate surfaces of the pure oxides (γ-Al_2_O_3_ and CeO_2_), slab models were created from the respective bulk oxide unit cells with an appropriate number of layers and a sufficient vacuum space of 15 Å in the direction perpendicular to the surface. The bottom-half layers of the surfaces were frozen during the structural optimizations to represent the bulk phase. The details of the bulk and surface structures along with their k-point grids are given in Tables S2 and S3, respectively.

## 3 Results

### 3.1 Reactions and Characterization of an Uncoated CZA40 Catalyst

**Figure 1:**
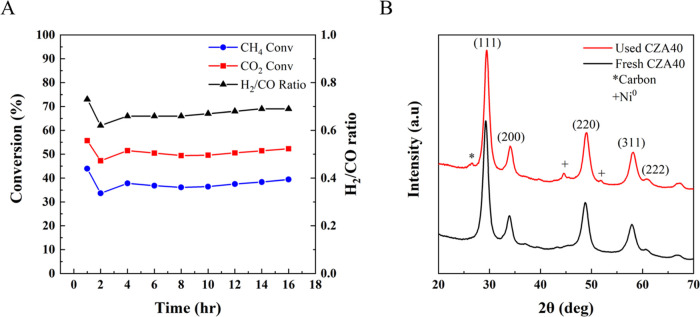
(A) DRM test of CZA40 (5 wt % Ni), 1:1 CH_4_/CO_2_ feed mixture at 750 °C, 1.2 bar, 37 000 GHSV. (B) XRD analysis of fresh and used CZA40.

Uncoated CZA40 (5 wt % Ni) was the reference material for this study. Initial characterization of fresh CZA40 was performed by XRD and N_2_ physisorption. The diffraction peaks (Figure 1) of the CZA40 powders were indexed to cubic CZA (ICSD 157416),^73^ with no observable peaks corresponding to Ni or NiO, indicating high dispersion of Ni in the fresh catalyst. The CeO_2_ crystallite size was calculated to be 5.5 nm for CZA40 based on the (111) reflection. The N_2_ physisorption (Table S4) gave a surface area of 95 m^2^/g by the BET method with a total pore volume of 0.64 cm^3^/g. Note the high surface areas maintained in the used catalysts.

CZA40 was tested in the fixed-bed reactor system at 750 °C and a gas hourly space velocity (GHSV) of 37 000 mL/(h·gcat). The catalyst exhibited initial conversions of 55% for CO_2_ and 44% for CH_4_ with an H_2_/CO ratio of 0.75, as shown in Figure 1. But at 16 h time onstream, an increase in reactor pressure to 1.7 bar was seen, indicating the presence of large quantities of carbon (coke) beginning to block the reactor. The final conversions were 52% for CO_2_ and 39% for CH_4_, with an H_2_/CO ratio of 0.69, but upon inspection, the reactor was nearly blocked. The low H_2_/CO ratio indicates a significant RWGS rate.

XRD analysis of CZA40 (Figure 1) revealed the emergence of two additional crystalline phases. The peak at 26.5° is indicative of semigraphitic carbon formation,^13^ while the peaks at 44.7 and 51.8° suggest large Ni^0^ aggregates.^13^ The calculated CeO_2_ crystallite size was 6.6 nm based on the (111) reflection; the increase in CeO_2_ crystal size reflects support sintering. The presence of both Ni^0^ and carbon phases confirms that the catalyst was being structurally altered, and while these changes did not deactivate the catalyst at first, they were sufficient to reduce its selectivity and lead to a situation in which the process became untenable.

### 3.2 Reactions on ALD-Coated CZA40 Catalysts

The TGA/DSC screening method at low CH_4_/CO_2_ conversions was used to determine short-term effects on activity/stability; this method was adopted unchanged from previous work.^3,13^ Results for all catalysts are listed in Table 1. The neglect of the effects of RWGS on the DSC heat flux is justified both by its relative thermoneutrality, and the knowledge that RWGS rates are always less than DRM rates (by an order of magnitude at low conversion, an assertion tested later).^13^ For example, the calculated endothermic heat of reaction for DRM is 7.2–7.5 times that of RWGS over 650–800 °C.^61^ The coking rate was determined by weight change over 1.5 h, observed after the first 0.5 h of reaction. The addition of the Al_2_O_3_ overlayer at a nominal 0.5 nm thickness did increase the reforming rate at 750 °C, but little to no benefit was seen at 650 °C. ALD of TiO_2_ significantly decreased the activity of CZA40 and was not investigated further.

**Table 1:** Catalyst Performance Metrics of Ni-Doped CZA (CZA40) and Ni-CZA Coated with Al_2_O_3_^a^

catalyst	DRM rate, 750 °C	DRM rate, 650 °C	coking rate, 750 °C	coking rate, 650 °C	coking rate, high conversion^e^	Δ*E*_a_, DRM^b^ kJ/mol
CZA40	0.21	0.14	1.3 × 10^–2^	4.5 × 10^–3^	5.7 × 10^–3^	32
0.3 nm ALD-CZA40-PR^c^	0.26	0.15	N/A^d^	2.3 × 10^–2^	1.7 × 10^–3^	43
0.5 nm ALD-CZA40	0.23	0.12	1.5 × 10^–2^	N/A^d^		51
0.5 nm ALD-CZA40-PR^c^	0.36	0.15	N/A^d^	N/A^d^	4.6 × 10^–4^	69
1.2 nm ALD-CZA40-PR^c^	0.15	0.05	N/A^d^	N/A^d^	2.4 × 10^–4^	86
0.5 nm TiO_2_ ALD-CZA40	0.15	0.077	N/A^d^	N/A^d^		52

a DRM Rate in mmol/(mg cat·h). Coking Rate in mg Coke/(mg cat·h). The coking rate was measured at >0.5 h into the run, but in some cases, the weight was still decreasing, indicating an essentially zero rate of coking.

b Observed activation energy (Arrhenius equation) for dry reforming.

c PR is prereduced at 550 °C with 5% H_2_/N_2_ for 3 h, prior to ALD.

d No measurable coke over 0.5–2 h time onstream.

e Computed from experiments below in the reactor system.

Prereducing 0.5 nm ALD-CZA40 (550 °C, 5% H_2_/N_2_, 3 h) prior to ALD was also investigated. These samples are shown as “PR” in Table 1, and the same pretreatments just prior to TGA were used. Prereduction prior to ALD greatly increased the DRM rate for 0.5 nm ALD-CZA40 at 750 °C, with a negligible coking rate at 750 °C compared to a nonpre-reduced ALD sample. Therefore, all other ALD-coated samples were also prereduced before ALD.

The DRM activity at 750 °C was comparable to uncoated CZA40 for the 0.3 nm sample, while the 1.2 nm sample had a lower activity. Both showed negligible coking rates at 650 and 750 °C. All three ALD-coated catalysts were further tested at high conversion in a bench-scale reactor system, as described below.

**Figure 2:**
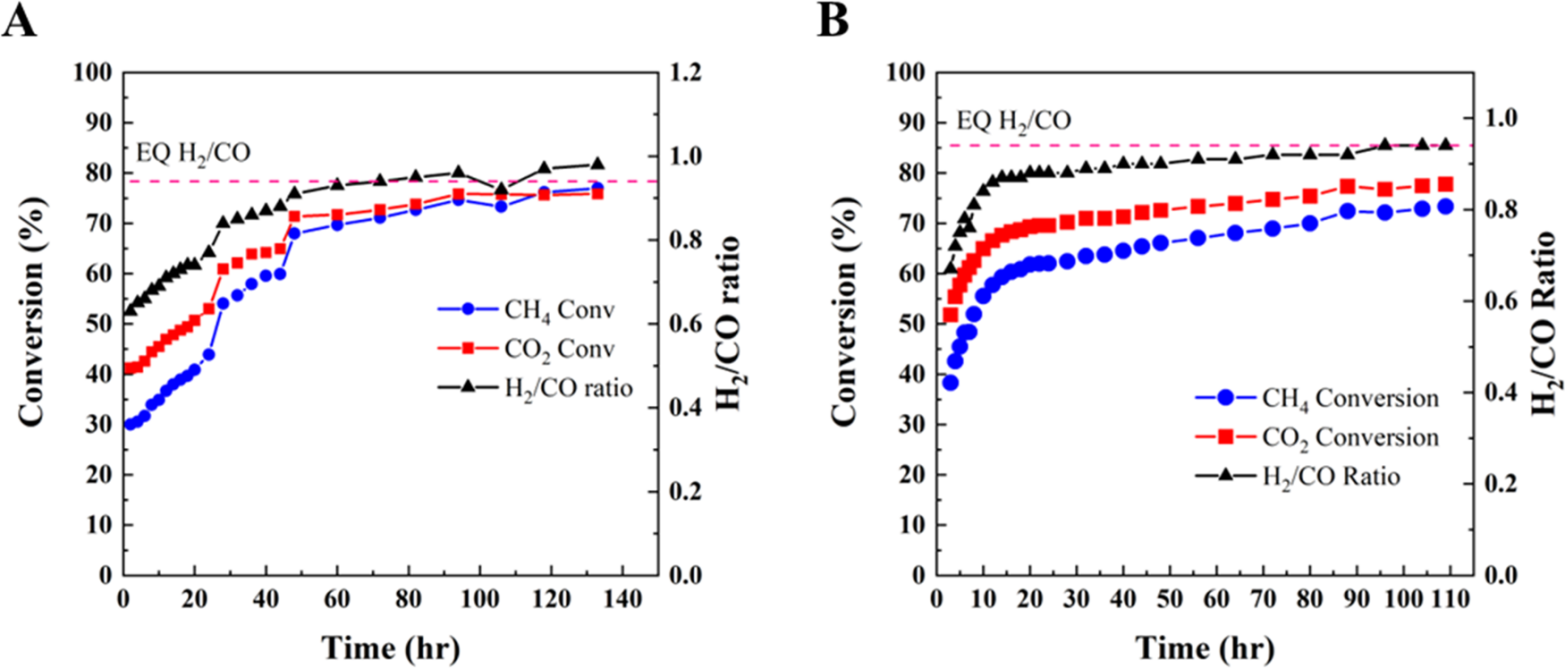
DRM test of (A) 0.5 nm ALD-CZA40 and (B) 0.3 nm ALD-CZA40 1:1 CH_4_/CO_2_ feed mixture at 750 °C, 1.2 bar, 10 900 GHSV. Equilibrium CO_2_ conversion is 94% and equilibrium CH_4_ conversion is 90%, calculated by Aspen HYSYS.

The 0.5 nm ALD-CZA40 was first tested at GHSV 10 900 mL/(h·gcat) at 750 °C (Figure 2). The sample was activated for more than 50 h before reaching its maximum activity and H_2_/CO ratio. After this time onstream, it exhibited ∼75% CH_4_ conversion with an H_2_/CO ratio of 0.98. This H_2_/CO ratio is near the equilibrium H_2_/CO ratio calculated using an Aspen HYSYS simulation of the process, including only the DRM and RWGS reactions. The run was continued to near 140 h time onstream, with no deactivation seen. The GHSV was then increased to 37 000 mL/(h·gcat) and the reactor was run for another 4 h (Figure S5). While the CH_4_ conversion and H_2_/CO ratio initially showed a slight decrease, the catalyst eventually attained nearly identical conversion and selectivity as at 10 900 GHSV. Comparing these results to uncoated CZA40 in Figure 1, the 0.5 nm ALD overcoat enhanced DRM activity and selectivity, increasing CH_4_ conversion from 40 to 77%, and H_2_/CO ratio from 0.7 to 0.98.

The total amount of coke deposited during DRM was estimated by temperature-programmed oxidation with averaged coking rates shown in Table 1. For the coated samples these can be an order of magnitude lower than the uncoated CZA40 sample. The TPO plots are in the Supporting Information (Figure S6).

The 0.3 nm ALD-CZA40 catalyst was also tested in the reactor system at 37 000 GHSV (Figure 2b). The catalyst’s induction period was ∼24 h, and it ran for 110 h with no deactivation. The final CH_4_ conversion was 74% at 0.94 H_2_/CO ratio, values only slightly below that of 0.5 nm ALD-CZA40.

### 3.3 Effect of ALD Overlayers on RWGS

The effects of Al_2_O_3_ ALD overlayers on RWGS were studied using a TGA/DSC method similar to the DRM low-conversion screening method of Table 1, at partial pressures (bar) 0.26 H_2_, 0.26 CO_2_, and 0.48 N_2_. The product of the H_2_ and CO_2_ partial pressures in this experiment is near the maximum possible calculated for the bench-scale reactor (0.068 bar^2^ here vs 0.09 bar^2^ for the bench-scale reactor at 33% fractional conversion), so this is a near-worst-case test for the undesired RWGS reaction. The heat flow values measured by DSC are highly characteristic of the RWGS reaction. Aspen HYSYS was used to simulate the equilibrium conversions and heat flows of RWGS at high CO_2_ and H_2_ partial pressures, along with its main competing reaction, CO_2_ hydrogenation (Sabatier reaction). At 650 °C RWGS contributed 95% of the total heat flow and at 750 °C 99.5% of the total heat flow. Thus, it can be assumed that CO_2_ hydrogenation to CH_4_ is negligible in these TGA/DSC experiments. Using the heat flow measurements and Aspen HYSYS, we calculated the H_2_ conversions for the TGA/DSC experiments were calculated. These H_2_ conversions were used to compute rate constants from a plug-flow reactor mass balance for CO_2_, for an assumed second-order RWGS reaction:
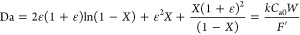
9where Da is the Damkohler number, a ratio of reaction rate to convective mass transport rate, ε the volumetric expansion factor (here, 0), *F*′ the total volumetric flow rate, and *X* is the fraction conversion.

The addition of the 0.5 nm ALD overlayer to CZA40 decreased the RWGS rate constants (Table 2) and increased the DRM rate constants that were computed from the differential rates of Table 1, assuming that the reaction is first-order in both CO_2_ and CH_4_. The present results showing that the addition of the Al_2_O_3_ overlayer to CZA40 decreased the RWGS rate constant are consistent with the H_2_/CO ratios near one that were observed in the high conversion reactor tests.

**Table 2:** Second-Order DRM and RWGS Rate Constants of Catalysts from Differential Rate Measurements, mL^2^/(min·mg_cat_·mmol)

catalyst	*k*_DRM_, 750 °C	*k*_DRM_, 650 °C	*k*_RWGS_, 750 °C	*k*_RWGS_, 650 °C	*k*_DRM_/*k*_RWGS_, 750 °C
CZA40	3.2 × 10^4^	1.6 × 10^4^	3.7 × 10^4^	3.2 × 10^4^	0.86
0.5 nm ALD-CZA40	6.1 × 10^4^	2.5 × 10^4^	2.3 × 10^4^	1.4 × 10^4^	2.7
0.5 nm ALD-CZA40, used^a^			2.4 × 10^4^	1.7 × 10^4^	

a 140 h time onstream DRM.

### 3.4 Characterization of ALD-Coated CZA40

Both fresh and used catalyst characterizations were preferentially performed on 0.5 nm ALD-CZA because it showed the highest activity.

#### 3.4.1 TPR, XRD, N_2_, and CO_2_ Adsorption

The CO_2_ DRIFTS experiments were performed on fresh CZA40 and 0.5 nm ALD-CZA40 catalysts to explore the integrity of the overlayer (Figure S7). As expected, the fresh CZA40 showed carbonate peaks and CO/CO_2_ masses (by MS) during desorption up to 400 °C. After the addition of the 0.5 nm shell and calcination, the carbonate signature was no longer detected. However, low concentrations of CO/CO_2_ could still be detected in the MS data, indicating minimal adsorption of CO_2_ on the coated surface.

**Figure 3:**
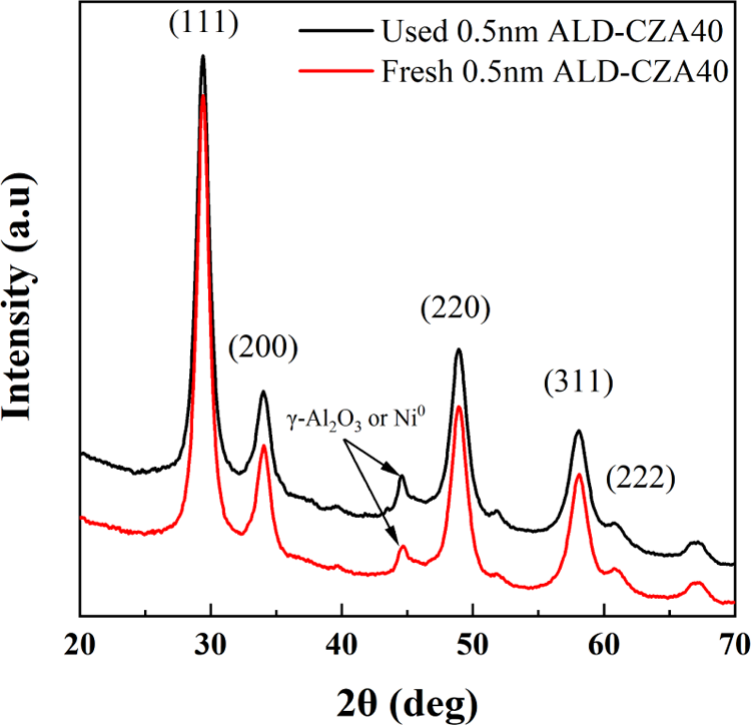
XRD spectra of used (red) and fresh (black) 0.5 nm ALD-CZA40.

Figure S8 shows the XRDs of fresh ALD-CZA40 catalysts and Figure 3 shows the XRDs of 0.5 nm ALD-CZA40 only. All three fresh catalysts show the characteristic peaks for cubic CZA (ICSD 157416).^73^ The peak at 44.4° for the 0.3 and 0.5 nm ALD-CZA40 probably arises from γ-Al_2_O_3_ only, because it does not disappear or change in intensity upon use (Figure 3). The XRD of used 0.5 nm ALD-CZA40 showed no peaks around 26.5° 2θ, indicating a lack of semigraphitic or graphitic carbon on the used catalyst.

##### 3.4.1.1 TPR Was Used to Verify the Gradual Structural Change Occurring in the ALD-Prepared Catalysts during Use

**Figure 4:**
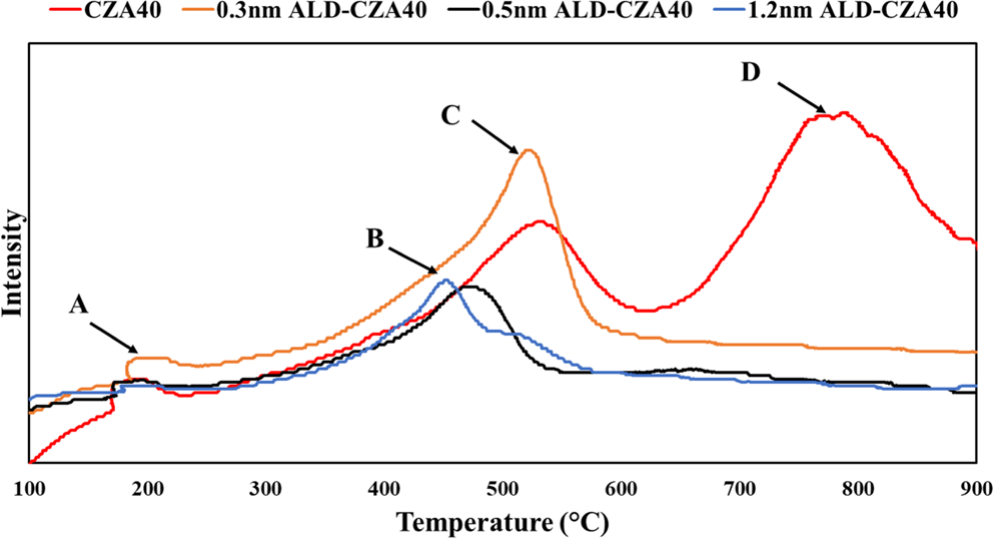
TPR analysis of the used 0.3 nm (orange line), used 0.5 nm (black line), used 1.2 nm (blue line) ALD-CZA40, and fresh CZA40 (red line).

Figure 4 compares the TPR analysis of used ALD-coated samples with the fresh CZA40 (the used sample was too coked to obtain meaningful TPR). There are four distinct peaks or shoulders. Peak D is attributed to bulk CeO_2_ reduction in CZA,^74−77^ and this peak disappears when the ALD overlayer is present, so all bulk CZA that could be reduced was reduced before or during reaction. Peak A is usually attributed to OV formation in doped Ni/CeO_2_,^20,76−79^ and note that it is largest in CZA40 and decreases substantially as the layer thickness increases, essentially disappearing for 1.2 nm ALD-CZA40. Peak C with a maximum of ∼530–535 °C is in the range (or below it) of a typical reduction peak for surface Ce with low loading Ni present, in 1:1 or near 1:1 molar CZA.^77,80−89^ The reductions of the two elements are intertwined. It is evident that under reaction conditions, the Ni and the Ce were not completely reduced. There are also contributions from surface or nanoparticle NiO at lower temperatures, which can be identified as the broad shoulders present between 300–430 °C.^20,78,84,86,88,90^ Ni^2+^ reduction within disordered, mixed CeO_2_–Al_2_O_3_ also shows a peak in this temperature range.^91,92^ Any Ni present as nanoparticle NiO reduces at least 50 °C lower than 530 °C.^81,86,88^ Any Ni intercalated within Al_2_O_3_ alone would reduce at higher temperatures, >580 °C.^93,94^

As the layer thickness increases, the area of peak C decreases and it appears only as a shoulder on B. The peak maximum shifts to a lower temperature, ∼480 °C for the 1.2 nm and 505 °C for the 0.5 nm ALD-CZA40. This shows that a different surface structure has been formed, which is neither entirely Al_2_O_3_ nor CZA. There are no high-temperature peaks in the ALD catalysts that would indicate the reduction of NiAl_2_O_4_,^91,94^ proving it is not formed during the reaction. It is notable that the 0.5 nm catalyst is the only one whose reduction is concentrated in a single broad peak.

The N_2_ adsorptions (Table S4) showed a decrease in surface area and pore volume for the ALD-coated samples compared to those of CZA40, at all coating thicknesses. The average pore diameters also decreased by more than 10%. These results are indicative of the ALD coatings penetrating pores, coating some and blocking some others, as has been previously observed for ALD-coated catalysts.^51,53^

#### 3.4.2 Ce XPS, Ni K-Edge XAS

XPS was used to explore the oxidation state of Ce, Ce 3d spectra showing multiple bands from 880–920 eV due to the O 2p valence band – Ce 4f hybridization. The deconvolution of Ce 3d spectra results in ten bands, six of which correspond to a Ce^4+^ state: *v* (884.8 eV), *v*″ (891.07 eV), *v*‴ (898.4 eV), *u* (903.2 eV), *u*″ (909.55 eV), *u*‴ (918.44), while the remaining four correspond to a Ce^3+^ state: *v*_0_ (881.4 eV), *v*′ (887.71 eV), *u*_0_ (900.32 eV), *u*′ (906.27 eV).^3,59,95^ The fraction of surface Ce^3+^ was estimated as
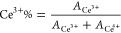
where *A*_Ce_^3+^ and *A*_Ce_^4+^ are the sums of the areas of the Ce^3+^ and Ce^4+^ peaks listed above.^96^ The spectra and deconvolutions applied are shown in Figure S9. The spectra of CZA40 and the fresh 0.5 nm ALD-CZA40 were similar, both with the percentage of surface Ce^3+^ calculated as 35% (of total surface Ce). The used 0.5 nm ALD-CZA40 showed a reduction in the 884.7 (*v*) band intensity, while increasing intensities are seen at 900.8 (*u*_0_) and 905.95 (*u*′). The surface Ce^3+^ for used 0.5 nm ALD-CZA40 is calculated as 71%, a factor of 2 increase over the fresh catalyst. Ce-based catalysts used under reducing conditions typically exhibit an increase in Ce^3+^.^97,98^ The amount of Ce^3+^ corresponds to the number of OV.^95,96,99^ A high concentration of surface OV is also associated with better coke resistance in Ni/CeO_2_ DRM catalysts.^3,21,34,100^ This is consistent with the DRM test results for both reactors. The Ce^3+^ percentages should be taken as relative only, due to the potential for XPS irradiation to create additional Ce reduction, but the exact magnitude of this machine-dependent reduction is unknown.^101^

O 1s was used to observe changes in the coordination environment of oxygen upon DRM (Figure S10). The O 1s spectra can be deconvoluted into 4 peaks: (1) organic species oxygen bonds associated with a 2^–^ formal charge including the double bond in C=O (O_1_) at ∼529 eV, (2) normal lattice oxygen (O_2_) at ∼532 eV, (3) lattice oxygen adjacent to vacancies (O_3_) at ∼534 eV, (4) oxygen in surface hydroxyl groups, adsorbed H_2_O, or surface carbonate at ∼537 eV (O_4_).^3,102−104^ There is a decrease in surface lattice oxygen from fresh to used 0.5 nm ALD-CZA40. This is consistent with the Ce 3d XPS results above showing an increase in undercoordinated surface Ce associated with Ce^4+^ reduction to Ce^3+^.

The XPS spectra were also used to estimate the surface and near-surface compositions of the ALD-CZA40 samples (Table 3). The coated samples are dominated by Al, suggesting the coatings are conformal. But there is some Ce and Zr, and these atomic %’s increase both as the coating gets thinner and as the catalyst is used in DRM. For the fresh catalysts, the Ce/Zr ratio is 1/2. But after 140 h of DRM the 0.5 nm ALD-CZA40 shows enrichment of (primarily) Ce on the surface, a 2.3% increase in Ce at %. The Ce/Zr ratio increased to 3/4 in the used sample, showing that Zr does not exhibit the same mobility during DRM as Ce.

**Table 3:** Atomic (At)% from XPS Spectra, Calculated on an Oxygen-Free Basis

sample	state	Al at %	Ce at %	Zr at %	Ce/Al ratio	Ce/Zr ratio
CZA40	fresh	81.7	6.1	12.1	1/13	1/2
0.3 nm ALD-CZA40	fresh	86.2	4.5	9.1	1/20	1/2
0.5 nm ALD-CZA40	fresh	87.4	4.2	8.3	1/25	1/2
	used	85.0	6.5	8.5	1/13	3/4
1.2 nm ALD-CZA40	fresh	90.6	3.0	6.3	1/33	1/2

**Figure 5:**
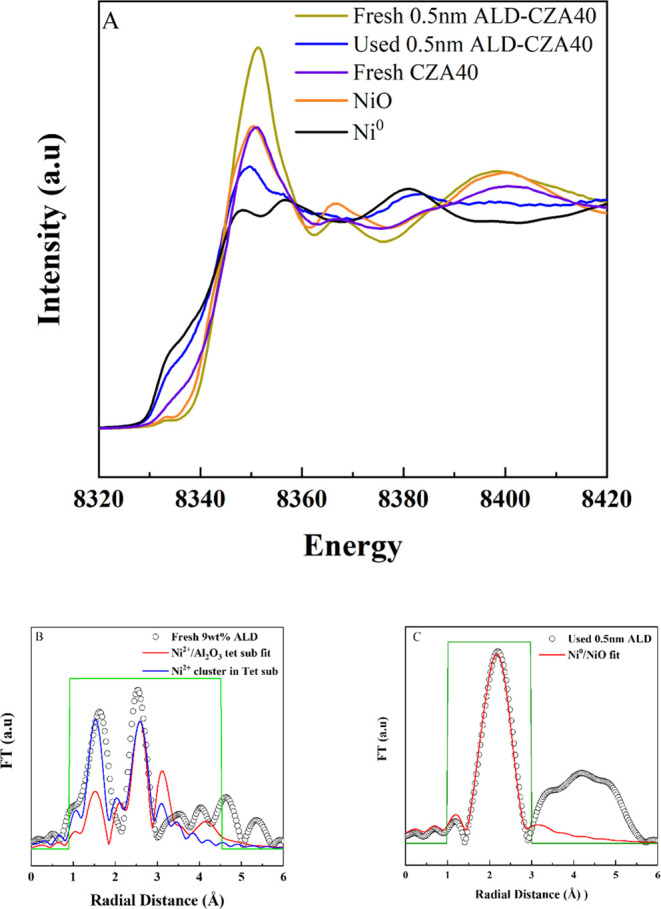
(A) Ni K-edge XANES spectra for CZA40, fresh, and used 0.5 nm ALD-CZA40. (B) Fourier transform (FT) XAFS for fresh 0.5 nm ALD-CZA40 (markers) compared to simulated curves for a single-atom Ni substitution in a tetrahedral site (red) and dual (“cluster”) Ni tetrahedral site substitution (blue). Green boxes are the fit range. (C) FT-XAFS for used 0.5 nm ALD-CZA40 (markers) compared to simulated curves for a Ni^0^/Ni^2+^mixture (red). Green box is the fit range.

XANES was used to determine the oxidation state of Ni in the same three catalysts as used for XPS. These Ni K-edge XANES are shown in Figures 5a, S11 and S12. The used 0.5 and 0.3 nm ALD-CZA40 catalysts clearly experienced reduction during DRM (Figures 5a and S12) and their XANES spectra are characteristic of a mixture of Ni^0^/Ni^2+^. XANES spectra of the used 0.3 and 1.2 nm ALD-CZA40 (Figure S11) show a greater degree of reduction than 0.5 nm to Ni^0^. Linear combination fitting on all three catalysts based on Ni foil and NiO standards gave a 65% Ni^0^ estimate for 0.5 nm ALD-CZA40 but a 90% estimate for the other two. Therefore, some Ni present in the ALD samples exists in the Ni^2+^ state, but there are subtle differences between these XANES spectra and that of a NiO standard. In comparison, fresh CZA40 shows some Ni^0^, but it is mostly Ni^2+^ as evident from the white line position at 8351 eV.

XAFS analyses were performed to further explore the coordination environment around the Ni. While XAFS fitting of Ni-CZA systems is documented,^59,105^ the addition of the overlayer and the 2+ charge on some of the Ni adds extra layers of complexity due to the unknown positions of Ni^2+^ atoms in either Al_2_O_3_, CeO_2_ (with ZrO_2_), CeAlO_3_, or as a Ni-containing phase such as NiAl_2_O_4_ or NiO. In particular, γ-Al_2_O_3_ has two types of octahedral and one type of tetrahedral site, in any of which Ni^2+^ could insert. To generate potential scattering pathway files in the Feff program, ATOMS files containing the positions of atoms and bond lengths for all of the above phases were obtained from XRD data, and then a single Ni core absorber was substituted for a cation in these files, except for NiAl_2_O_4_ and NiO, where the Ni atoms were already present.^106−109^ The numbers of oxygen atoms were adjusted to maintain electroneutrality by removing an O atom within the scattering volume, which consisted of a sphere of radius 5 Å from the core absorber. The XAFS analyses of used 0.3, 0.5, and 1.2 nm ALD-CZA40 proved less difficult due to the much larger concentrations of Ni^0^ present in these samples. For XAFS analysis of the used 0.5 nm ALD-CZA40, the data were regressed by weighting the *S*_0_^2^ values of pure Ni and NiO phases: only the first-shell [Ni]–Ni, [NiO]–O, and [NiO]–Ni paths were included. This multiphase process is consistent with past work on first-shell fits of mixed Ni^0^/Ni^2+^ in oxides.^59^

Of all the possible phases listed above, only NiO itself (Figure S14) and Ni^2+^ substituted into a tetrahedral γ-Al_2_O_3_ site (Figure 5b) gave reasonable fits for the fresh catalyst (the fits are given in Table S5, and the poorer fits for Ni/CeO_2_ and NiAl_2_O_4_ are shown in Figures S13 and S15). This conclusion was based on both visual observation and the relative standard deviations of the FT-XAFS functions (the *R*-values). The fit to NiO is not exact, but that is expected due to the complex nature of the coated samples. It is highly unlikely that the Ni is in bulk NiO, which would be inconsistent with the XRD results and the known reduction chemistry of NiO, but the XAFS fits do suggest a coordination environment characterized more by adjacent Ni and O atoms in the fresh sample, consistent with the XANES.

Figure 5b shows the fit for a single Ni^2+^ atom substituted into a tetrahedral γ-Al_2_O_3_ site for fresh 0.5 nm ALD-CZA40. The peak at 1.5 Å arises from first-shell O atom scattering. The nature of the peak at 2.5 Å cannot be so easily determined due to potential scattering from Ni, Al or Ce atoms, all of which could be located near 2.5 Å radial distance. To analyze the nature of this peak a new ATOMS file based on γ-Al_2_O_3_ was constructed with a second Ni atom placed in a tetrahedral site adjacent to the Ni core absorber. Electroneutrality was again maintained by removing oxygen atoms, as necessary. Comparisons of fresh 0.5 nm ALD-Ni-CZA to Feff simulations of both single and dual Ni-atom substituted γ-Al_2_O_3_ are shown in Figure 5b. The dual Ni atom simulation conforms better to the experimental data. The *R*-factor decreases by roughly half (Table S6) upon adding the second Ni atom. It is evident that additional Ni atoms could be added at either tetrahedral or octahedral sites, probably further improving the fit, but the number of permutations in Ni siting becomes almost infinite as the number of Ni atoms in Al_2_O_3_ cation vacancies increases. The significant results from the XAFS of the fresh catalysts are that (a) there is Ni–O clustering and (b) the clustering is more likely in the alumina overlayer and not in a pure CZA.

XAFS was also used to estimate Ni cluster sizes in the used catalysts. XAFS analysis of used 0.5 nm ALD-CZA40 is shown in Figure 5c. The largest peak can be assigned to the Ni–Ni scattering of Ni^0^. However, this peak is broader when compared to samples with less Ni^2+^ present, such as the used 0.3 and 1.2 nm ALD-CZA40 (Figures S16a,b). This suggests that additional scattering paths give rise to this peak. As previously mentioned, a weighted Ni^0^/Ni^2+^ fit was applied by weighting the *S*_0_^2^ according to the mole fraction of Ni^0^ and NiO. The mole fraction was a regression parameter. The coordination number for the Ni–Ni scattering path of this fit is much less than in either NiO (*N* = 6 for O, 12 for Ni) or Ni foil (*N* = 12), suggesting that Ni here exists as dispersed clusters, where much of it is coordinatively unsaturated (Table S6). This type of behavior has been observed previously, with the extent of coordinative unsaturation suggesting clusters of average size less than 3 nm.^59,105,110^

#### 3.4.3 High-Angle Annular Dark Field Scanning Transmission Electron Microscopy (HAADF-STEM) and EDX

**Figure 6:**
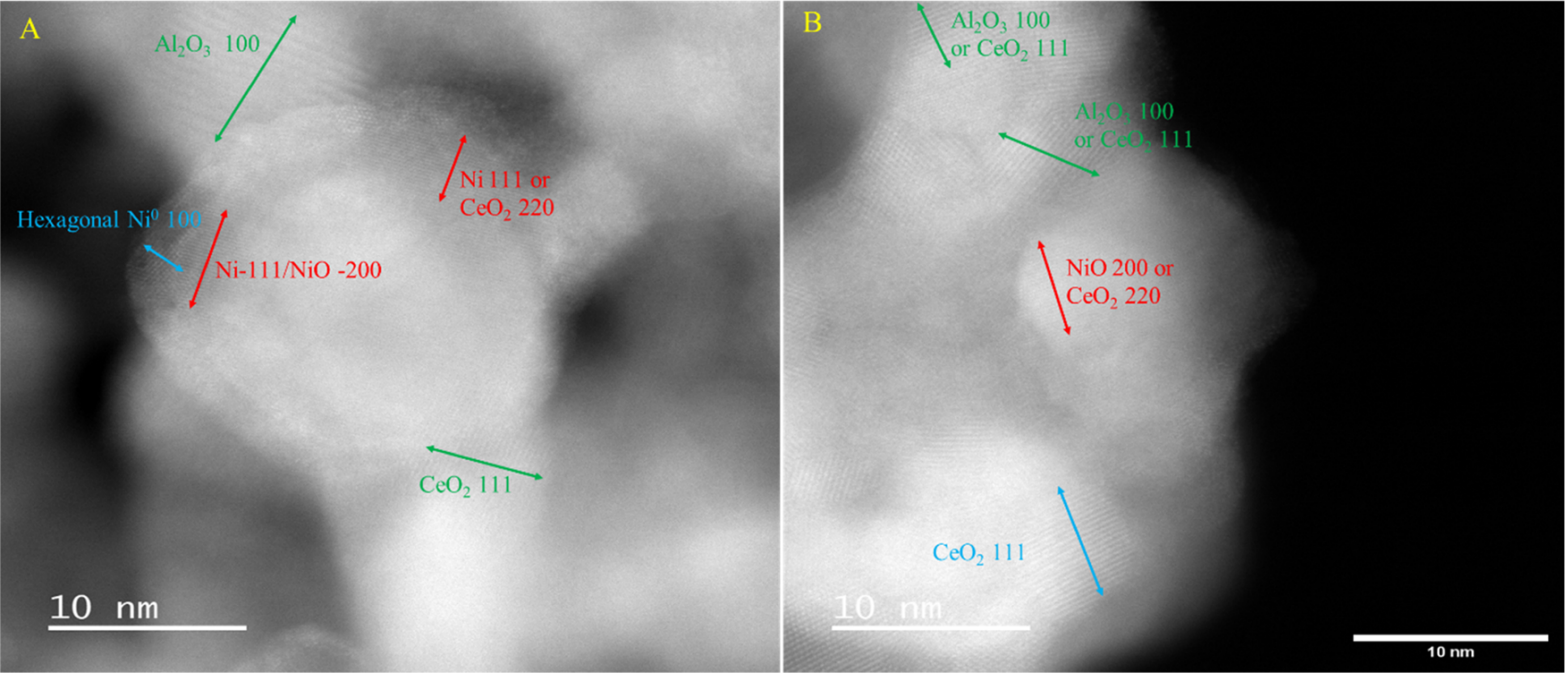
(A) DF STEM image of fresh 0.5 nm ALD-CZA40 and (B) DF STEM image of used 0.5 nm ALD-CZA40.

These experiments confirmed several of the conclusions from other characterization results. Dark-field STEM and EDX images of fresh and used 0.5 nm ALD-CZA40 samples were obtained. In Figures 6a,b and S17–S19 the planes marked CeO_2_ represent a CZA with a distorted fluorite structure, consistent with the XRD results (not all such CeO_2_ regions are marked in the figure). It is impossible to distinguish the (200) plane of cubic NiO or the (111) plane of cubic Ni (0.203 nm) from the (220) plane of CZA (0.191 nm CeO_2_, 0.186–0.192 nm for CZA), making it difficult to assign some lattice fringes to either species, thus these fringes are marked as representing Ni, NiO or CeO_2_ planes. All of these planes are common in their respective species. Planes with *d* spacings around ∼0.221 nm are assigned to the (100) plane of a hexagonal Ni phase. No carbon filaments, typically seen on heavily coked DRM catalysts,^111^ are seen on the used 0.5 nm ALD-CZA40. But there are also regions characteristic of an Al_2_O_3_ phase in Figures 6a,b and S19b. It was impossible to perform this analysis for uncoated, used CZA40 (Figure S20) given the large amount of surface coke. The DF STEM images illustrate the complex semicrystalline environment of the Ni and suggest why it is difficult to get exact fits to model structures in EXAFS.

**Figure 7:**
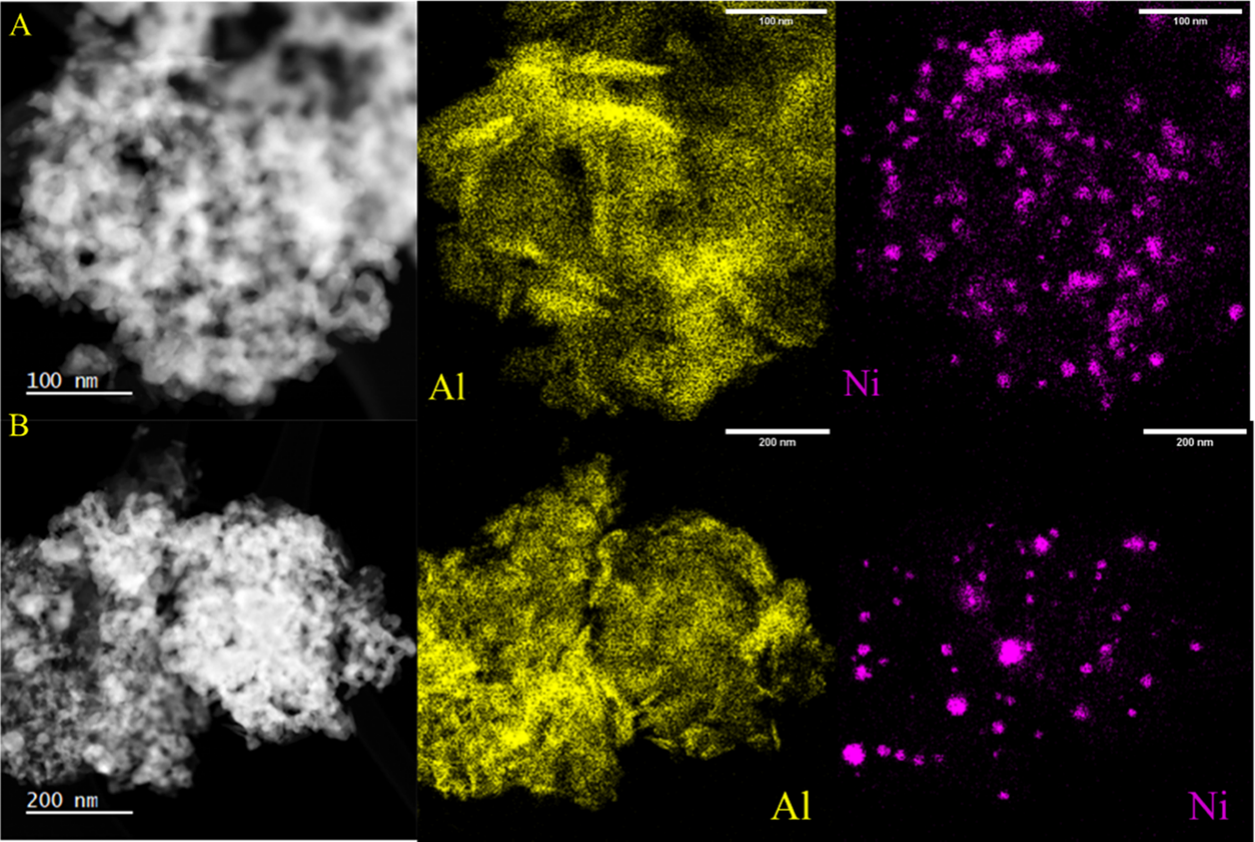
(A) EDS mapping for a DF STEM image of fresh 0.5 nm ALD-CZA40. (B) EDS mapping for a DF STEM image of used 0.5 nm ALD-CZA40.

Figure 7a,b shows EDX maps of both fresh and used 0.5 nm ALD-CZA40. Both images are at lower magnification, capturing several Ni particles. The fresh sample (Figure 7a) shows dispersed Ni and no segregation of Al. The used sample (Figure 7b) clearly lost some of its initially high Ni dispersion due to crystal ripening, but there are still many regions of dispersed Ni present. An average surface Ni particle size was obtained from the EDS images for both fresh and used 0.5 nm ALD-CZA40. Upon use this average particle size increased from 15.8 to 17.3 nm, consistent with a slight loss of Ni dispersion, but given the limited resolution, this population is only characteristic of the largest Ni clusters or grouping of clusters.

### 3.5 Insights into DFT Calculations

This section is divided into four subsections. Section 3.5.1 discusses the thermodynamic feasibility of forming ceria-Al_2_O_3_ mixed oxides in the bulk and surface. The stability and reducibility of Ni-incorporated ceria are compared to pure ceria surfaces in Section 3.5.2, along with calculations supporting the favorability of Ce^4+^ reduction post Ni incorporation, as observed in both the TPR and XPS studies. Section 3.5.3 provides information on the thermodynamic stability of Ni–Al_2_O_3_ bulk structures, in agreement with the XAFS fitting results. The stability of the Ni–Al_2_O_3_ surfaces is analyzed to gauge the extent of mixing of Ni with an Al_2_O_3_ overlayer. Section 3.5.4 presents calculated stability comparisons between Ni-ceria and Ni–Al_2_O_3_ surfaces to comment on the location preferences of small Ni clusters or isolated Ni atoms.

#### 3.5.1 Thermodynamic Feasibility of Ceria-Al_2_O_3_ Mixing

To understand the overlayer mixing of CeO_2_ with Al_2_O_3_, DFT results are presented exploring the thermodynamic feasibility of forming both bulk and surface ceria-Al_2_O_3_ mixed oxides.

##### 3.5.1.1 Bulk Mixing of Ceria-Al_2_O_3_

γ-Al_2_O_3_, Ce_2_O_3_, and CeAlO_3_ bulk oxides were used as parent structures in which Ce was isovalently substituted for Al or vice versa (referred to as doping). Doping with Ce^4+^ was not examined because aliovalent substitution of Ce requires the insertion of additional O or OH at the substituted positions. The formation energies of the doped oxides, referenced to their bulk oxide precursors, were normalized by the number of Ce and Al cations in the structure for accurate comparison among structures, as shown below:
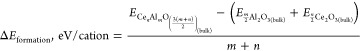
10*E*_*j*_ is the DFT energy for species *j* within the reaction:
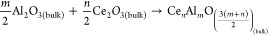
11where *m* and *n* are stoichiometric coefficients. Structural DFT energies of the doped oxides were minimized with respect to the volume of the unit cell, to account for the lattice distortion postdoping (details in the Supporting Information). The Ce concentration (cation %) in each bulk oxide is calculated according to eq 12.
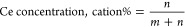
12

Figure S21 shows per cation formation energies for these Ce–Al mixed oxides as a function of the Ce concentration. Formation energies of all Al-doped Ce_2_O_3_ structures were much greater (by >1 eV/cation) than other structures of overall similar Ce concentration and are therefore not presented for discussion. Formation energies of all Ce-doped γ-Al_2_O_3_ oxides (blue squares, Figure S21) are positive (∼0.15 and 0.4 eV/cation), indicating that these are less stable than the segregated parent oxides. These data, together with an expansion of unit cell volume upon Ce doping (>2% volume increase), reveal the significance of lattice strain induced due to Ce doping; the larger ionic radius of Ce^3+^ compared to that for Al^3+^ (1.15 vs 0.68 Å) renders these doped structures unfavorable. Substitution of Ce into tetrahedrally coordinated positions in the γ-Al_2_O_3_ lattice (Al–O ∼ 1.8 Å in parent structure) compared to octahedrally coordinated positions (Al–O ∼ 1.95 Å in parent structure) resulted in both smaller unit cell expansion (1.9 vs 2.5%) and a lower formation energy (+0.16 vs +0.20 eV/cation).

Cerium aluminate (CeAlO_3_) is a stable bulk mixed oxide relative to its parent oxides. Note here that we do not observe this bulk oxide formation in our experimental work, but it is observed in other works.^112−114^ A few nonstoichiometric cation substitutions into this mixed-oxide phase have favorable formation energies (Ce_(0.44–0.53)_Al_(0.47–0.56)_O_3_). However, these mixed-oxide phases are not stable relative to phase separation as their formation energies do not fall on the convex hull. Together, these results indicate that the formation of mixed Ce–Al bulk oxides is unfavorable, with the exception of the stoichiometric CeAlO_3_ phase, for which there is no experimental evidence.

##### 3.5.1.2 Surface Doping of Ce on Al_2_O_3_

Cell volume expansion can be negligible upon surface-level doping; therefore, doping of Ce on γ-Al_2_O_3_ surfaces was examined to assess the stability of the experimentally observed mixed CeAlO_*x*_ overlayers. The two most stable facets of γ-Al_2_O_3_, (110) and (100) were examined (refer to Figure S22). Single/multiple Al sites that are highlighted with yellow circles in Figure S22c and d were substituted with Ce. The oxidation state of doped Ce was varied from 3+ to 4+. Doping energy, in eV, is used to assess the thermodynamic stabilities of Ce-doped γ-Al_2_O_3_ surfaces (reaction equations in Tables S7 and S8).

**Figure 8:**
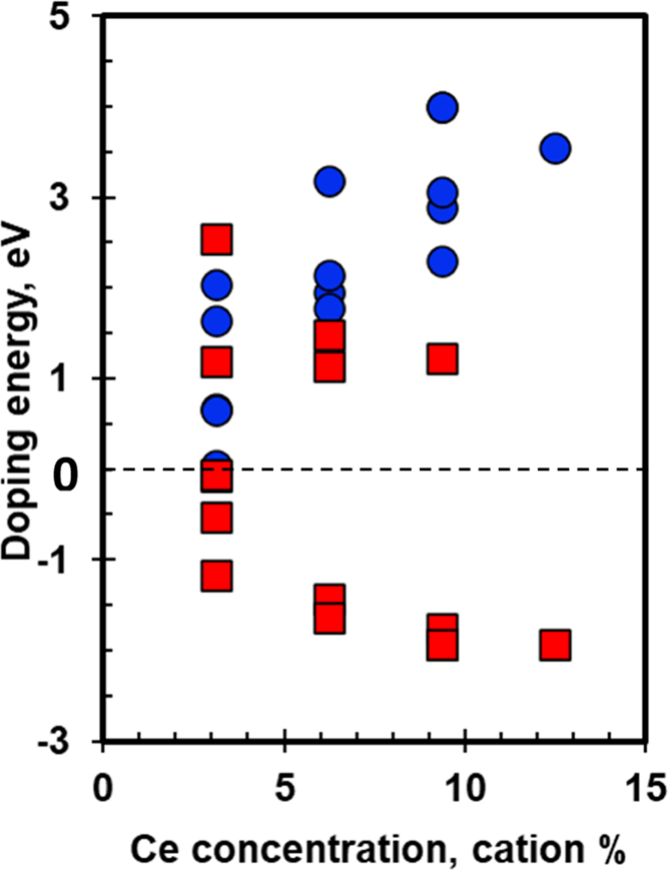
Doping energy as a function of Ce content for the Ce^3+^-doped γ-Al_2_O_3_ (100) (blue circles) and (110) (red squares) surfaces.

**Figure 9:**
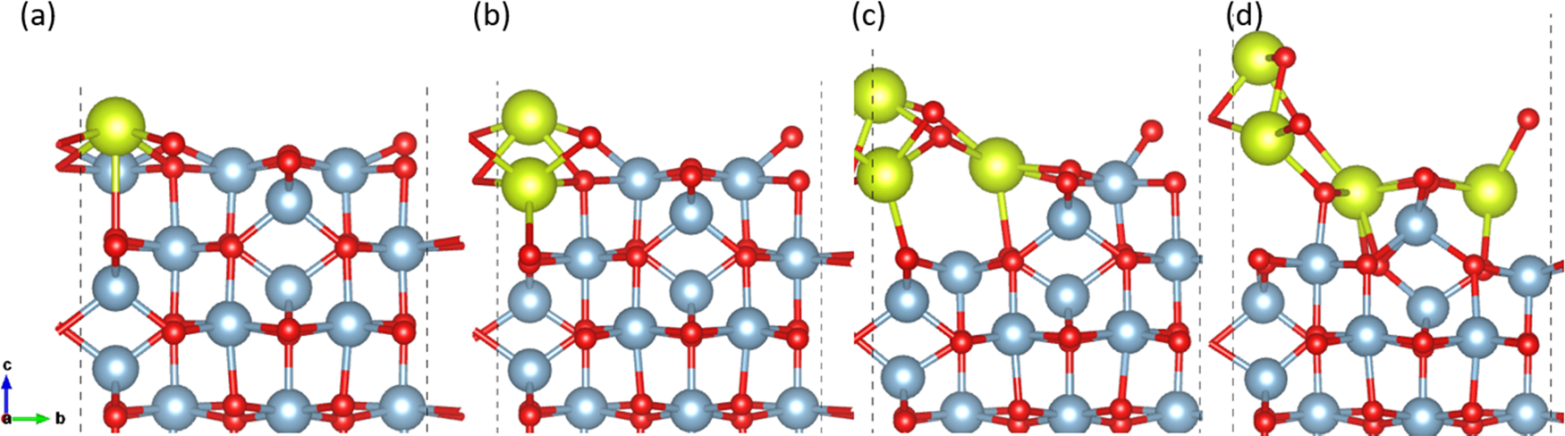
(a) 3.1% Ce^3+^ doping on the (100) facet (12 site), (b) 6.25% Ce^3+^ doping on the (100) facet (12, 13 sites), (c) 9.4% Ce^3+^ doping on the (100) facet (12, 13, 02 sites), and (d) 12.5% Ce^3+^ doping on the (100) facet (12, 13, 02, 08 sites). The yellow, light blue, and red spheres represent Ce, Al, and O atoms, respectively.

**Figure 10:**
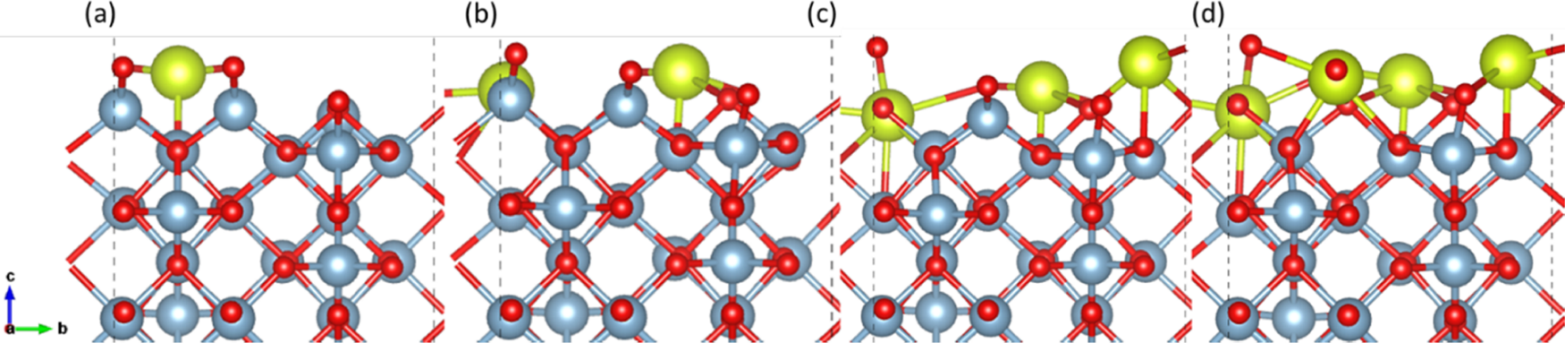
(a) 3.1% Ce^3+^ doping on the (110) facet (14 site), (b) 6.25% Ce^3+^ doping on the (110) facet (9, 14 sites), (c) 9.4% Ce^3+^ doping on the (100) facet (9, 14, 1 sites), and (d) 12.5% Ce^3+^ doping on the (100) facet (9, 14, 1, 7 sites).

Figure 8 shows the doping energy as a function of the Ce^3+^ concentration for γ-Al_2_O_3_ (100) and (110). Ce doping on the (100) facet becomes highly unfavorable with increasing Ce concentration, with the lowest doping energy at ∼3% Ce concentration (0.04 eV) being positive. Doping induces surface reconstruction, which intensifies with increasing Ce concentration. This can be observed in Figure 9, where the doped Ce atoms tend to detach from the surface by breaking their bonds with near-surface O atoms (Figure 9b,c,d). However, Ce doping on the (110) facet is relatively favorable, with the doping energies being negative for a few structures, even at higher Ce concentrations. Minimal surface reconstruction is observed postdoping, as showcased in Figure 10. Owing to its corrugated surface, with ∼45% higher surface area than the (100) facet, the (110) facet is more favorable toward Ce doping.

#### 3.5.2 Stability and Reducibility of Ni-Ceria Surfaces

Next, we compare the stability of Ni in adsorbed and doped configurations on CeO_2_ surfaces to further understand the reducibility of such mixed-oxide surfaces.

##### 3.5.2.1 Stability of Doped vs Adsorbed Ni-Ceria Surfaces

The most stable (111) facet of CeO_2_ (Figure S23) was chosen for this study. NiO was adsorbed on the CeO_2_ surface (“adsorbed”), or a Ni atom was substituted in place of a surface Ce along with the creation of an oxygen vacancy (“doped”). The doping and adsorption energies were calculated as the reaction energies given in eqs 13 and 14, respectively. A negative value of ΔΔ*E* (in eV/Ni) = Δ*E*_doping_ – Δ*E*_adsorbed_ for the doped configuration (Table 4) implies that Ni prefers to be doped rather than adsorbed. Despite an equivalent average Ni–O bond distance (∼2.01 Å) in both configurations, noteworthy distinctions in the coordination environment of Ni are observed. Specifically, in the doped structure, Ni achieves relatively symmetrical coordination characterized by each Ni–O distance being around 2.00 Å. In contrast, the adsorbed structure manifests a broader range of Ni–O distances, spanning from 1.96 to 2.10 Å, indicative of a less symmetrical coordination (Figure S24).

**Table 4:** ΔΔ*E* (eV/Ni) Calculation for the Ni-Doped (111) CeO_2_ Surface Referenced to Δ*E* of NiO-Ce_16_O_32_

configuration	reaction	ΔΔ*E* (eV/Ni)
adsorbed	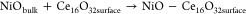 13	0.00
doped	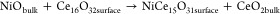 14	–2.07

##### 3.5.2.2 Oxygen Vacancy (OV) Formation Energetics of Ni-Doped and Bare Ceria Surfaces

Given the observation that Ni prefers to dope within rather than adsorb atop the ceria surface, the reducibility of Ni-doped CeO_2_ surfaces was evaluated by comparing the OV formation energies (Δ*E*_ovf_) of the doped and parent surfaces (eq 15). In both NiCe_15_O_31_ and Ni_2_Ce_14_O_30_, OVs were created by removing a Ni-bonded O atom, as this yielded the most stable structures. After the creation of the OV, Ni stays in a formal Ni^2+^ state, with two Ce^4+^ reducing to Ce^3+^ in both structures (formal charges are assigned based on the spin densities of individual atoms).
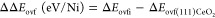
15

Table 5 shows ΔΔ*E*_ovf_ values for singly and doubly Ni-doped CeO_2_ surfaces. The doping of (111) CeO_2_ with a single Ni^2+^ atom results in a positive ΔΔ*E*_ovf_, taken relative to OV formation at the same concentration in the undoped (111) CeO_2_ surface. But doping of two Ni atoms into the CeO_2_ (111) surface promotes OV formation relative to the undoped surface (negative ΔΔ*E*_ovf_).

**Table 5:** ΔΔ*E*_ovf_ Calculation for Ni-Doped (111) CeO_2_ Surfaces (Referenced to Δ*E*_ovf, Ce16O32_)

surface	reaction equation	ΔΔ*E*_ovf_
(111) CeO_2_	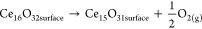 16	0.00 eV
1Ni^2+^ doped (111) CeO_2_	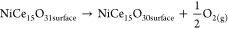 17	+0.38 eV/Ni
2Ni^2+^ doped (111) CeO_2_	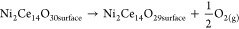 18	–1.67 eV/Ni

#### 3.5.3 Stability of Ni-Alumina Systems

Results from XANES and XAFS experiments indicate the presence of Ni^2+^ in γ-Al_2_O_3_ and the favorability of Ni clustering in the alumina lattice. This section presents results from DFT calculations of Ni–Al_2_O_3_ systems that support these experimental findings.

##### 3.5.3.1 Doping of Ni in Bulk Al_2_O_3_

**Figure 11:**
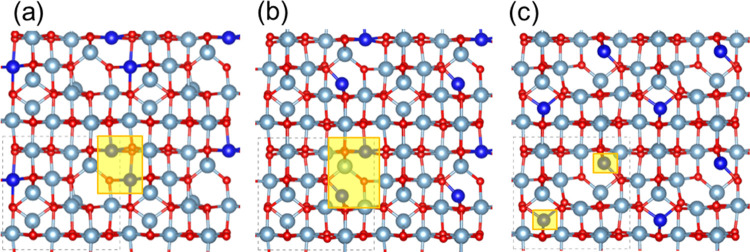
Two Ni^2+^ atoms doped into γ-Al_2_O_3_ with different site combinations (a) octahedral–octahedral, (b) octahedral–tetrahedral, and (c) tetrahedral–tetrahedral; the highlighted portions indicate the substituted Ni atoms.

Ni-doped γ-Al_2_O_3_ was generated by replacing two Al sites in the γ-Al_2_O_3_ bulk structure with two Ni atoms. The doped Ni atoms were forced to stay as Ni^2+^ by removing either a Ni-bonded or the nearest Al-bonded O atom. The doped site combinations used for this study are classified into three broad categories: octahedral–octahedral, octahedral–tetrahedral, and tetrahedral–tetrahedral (refer to Figure 11). Only the DFT energy of the most stable structure under each category was employed to calculate Δ*E*_formation_ and ΔΔ*E*_formation_ (Table 6). The Δ*E*_formation_ per Ni of the doped structures was calculated with reference to bulk γ-Al_2_O_3_ and NiO, as shown in eq 19. Here, ΔΔ*E*_formation_ indicates the preference of sites for Ni doping relative to doping in tetrahedral–tetrahedral sites (eq 20).
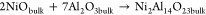
19
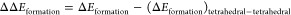
20

**Table 6:** Formation Energies of Ni^2+^-Doped γ-Al_2_O_3_ Bulk Structures (Referenced to the Δ*E*_formation_ of Ni Substituted in a Tetrahedral–Tetrahedral Site Combination)^a^

sites for two Ni-doped bulk γ-Al_2_O_3_	ΔΔ*E*_formation_ (eV/Ni)
octahedral–octahedral	–1.41
tetrahedral–octahedral	–1.10
tetrahedral–tetrahedral	0.00

a Ni^0^-doped γ-Al_2_O_3_ bulk structures were also simulated but gave highly positive formation energies relative to Ni^2+^-doped γ-Al_2_O_3_ (>+1 eV difference).

##### 3.5.3.2 Stability of Doped vs Adsorbed Ni–Al_2_O_3_ Surfaces

**Figure 12:**
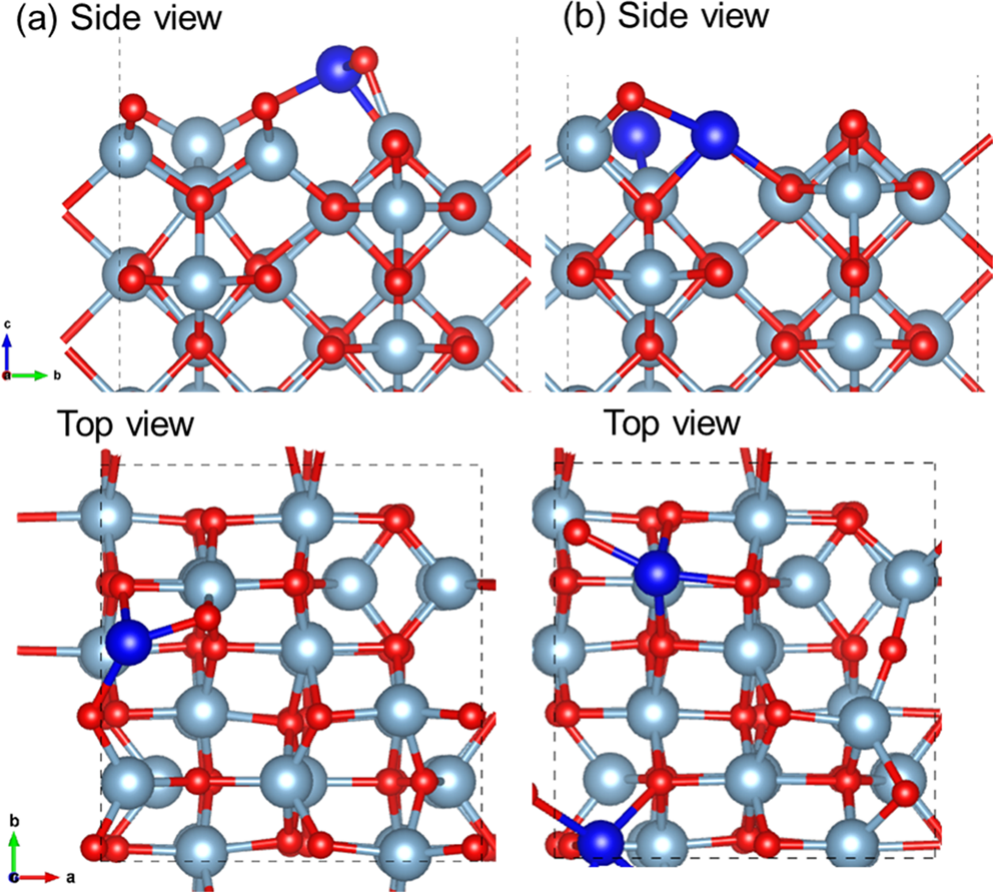
(a) Side and top view of the Ni^2+^-adsorbed (110) γ-Al_2_O_3_ surface and (b) side and top view of the Ni^2+^-doped (110) γ-Al_2_O_3_ surface (09 and 14 sites–doping sites).

Understanding the doping energetics of Ni on Al_2_O_3_ surfaces is crucial to understanding where Ni prefers to locate – on ceria or on Al_2_O_3_. Thus, the ΔΔ*E* of the most stable Ni^2+^-doped (110) γ-Al_2_O_3_ surface is reported here with reference to the most stable Ni^2+^-adsorbed (110) γ-Al_2_O_3_ surface (eq 21). Ni^2+^-doped (110) γ-Al_2_O_3_ surfaces were generated by replacing two surface Al atoms with two Ni atoms, followed by the removal of a Ni-bonded O atom. The structures are shown in Figure 12 and the results in Table 7 show that the doped structure is considerably more stable.
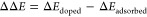
21

**Table 7:** ΔΔ*E* of Ni^2+^-Doped (110) γ-Al_2_O_3_ Surface (the Reference is the Δ*E* of NiO-Al_32_O_48_)

configuration	reaction	ΔΔ*E* (eV/Ni)
adsorbed	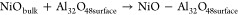 22	0.00
doped	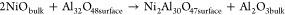 23	–1.04

#### 3.5.4 Preference for Ni–Al_2_O_3_ over Ni-Ceria Structures

To determine the relative stabilities of Ni-doped surfaces, we computed reaction energies for a two Ni^2+^-doped (110) γ-Al_2_O_3_ surface (Ni_2_Al_30_O_47_) and a two Ni^2+^-doped (111) CeO_2_ surface (Ni_2_Ce_14_O_30_). The Ni_2_Ce_14_O_30_ surface structure was chosen because it was the most stable structure with no surface Ce^4+^ reduced. DFT energies of the individual species in eq 24 were used to calculate the energy required to form Ni_2_Al_30_O_47_ relative to Ni_2_Ce_14_O_30_. A negative Δ*E* indicates that Ni has a strong preference for doping alumina rather than ceria.
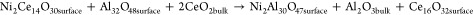
24
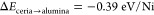


## 4 Discussion

### 4.1 Observations of the Kinetics

The rate constants calculated from the low conversion data are given in Table 2. The *k*_DRM_ for the fresh ALD-CZA40 catalysts can be used in eq 9 with other parameters charcteristics of DRM in the high conversion reactor (*W* = 250 mg, *F*′ = 150 mL/min, *C*_A0_ = 2.03 × 10^–5^ mol/mL) to predict an upper bound on conversion. It is an upper bound because the reverse rate constant and product/reactant inhibition would have to be considered to determine a more realistic conversion. However, the expected CH_4_ conversion in the larger reactor using *k*_DRM_ from Table 2 for 0.5 nm ALD-CZA40 was only 53%, compared to an actual CH_4_ conversion of 75%. This significant difference in expected vs actual conversion demonstrates the magnitude of the structural change of the ALD-coated catalyst, which is evolving to a far more active state than what was initially put into the reactor. This is evident as well from the long induction period (Figure 2). Induction periods in Al_2_O_3_ ALD-coated catalysts have been observed before and were also associated with structural evolution.^51,53^

The analysis of the rate constants of DRM and RWGS is also useful for understanding why the ALD-coated catalysts are more selective. With uncoated CZA40, the ratio *k*_DRM_/*k*_RWGS_ at 750 °C was slightly less than 1 while at 650 °C it was well below 1. For fresh 0.5 nm ALD-CZA40 the ratios at 750 and 650 °C were both greater than 1, with the ratio increasing with respect to temperature, as is generally true for good DRM catalysts. Both of these rate constants were obtained at conditions of equal partial pressures of the respective reactants for RWGS and DRM. For RWGS an equal ratio of partial pressures H_2_:CO_2_ is an oversimplification of the nature of these two reactants in a reactor, where the H_2_ and CO_2_ concentrations vary greatly with reactor length. At the entrance of the reactor, there is a lot of CO_2_ but no H_2_. Eventually, both H_2_ and CO_2_ are present and can react to H_2_O and CO by RWGS, although because of the nature of the DRM reaction, they are not likely to be equal in partial pressure. Further into the reactor, RWGS is less likely, because there is little CO_2_. The ALD-coated catalyst exhibited two desirable behaviors, the first being a decreased *k*_RWGS_ and the second being an increased *k*_DRM_, both in absolute terms. The larger *k*_DRM_ means more CO_2_ consumption, and as more CO_2_ is consumed the RWGS (which has an equilibrium constant of 0.48–0.76 at 650–750 °C) gradually reverses to water–gas shift, regenerating H_2_. Therefore, the change in both forward rate constants in favorable directions leads to the high H_2_/CO ratio observed for 0.5 nm ALD-CZA40.

### 4.2 Nature of Surface Ni–Al–Ce Interactions in DRM

Fully understanding the operando surface structure of 0.5 nm ALD-CZA40 or any other overcoated catalyst is a difficult to impossible task. However, with the characterization and DFT data presented here, it is possible to make hypotheses about the oxidation state and coordination environment of the surface Ni, Ce, and Al. From the K-edge Ni XANES and XAFS results we concluded that some of the Ni remained electropositive, and from all the XAFS fits, and in particular, by comparing FT-XAFS fits for two vs one atoms of Ni^2+^ occupying a tetrahedral alumina site (Figure 5b), we know that the most likely coordination environment for the electropositive Ni is Ni^2+^–O clustering. The Ni is not bulk NiO or surface-adsorbed NiO due to the lack of a visible NiO phase in XRD (Figure 3), the known propensity of NiO to reduce to Ni^0^ at DRM conditions,^115^ and the DFT calculations (Section 3.5.3.2) which reveal that a Ni^2+^-doped (110) alumina surface manifests a negative ΔΔ*E* (in eV/Ni) relative to adsorbed NiO. As a dopant, Ni^2+^ gets the benefit of attaining a coordination number closer to what it would have in bulk NiO. This validates the preference of Ni to dope rather than adopt the undercoordinated adatom configuration. Furthermore, a doping energy comparison between Ni-doped ceria and Ni-doped alumina surfaces revealed a thermodynamic preference for Ni to dope γ-Al_2_O_3_, with a minimum favorability of −0.39 eV/Ni (eq 24).

In the 0.5 nm ALD-CZA40 catalyst, more Ni remained embedded in this oxide lattice (which could also be a NiO-Al_2_O_3_–CeO_2_ mixture) during long-term DRM. While the tetrahedral site of γ-Al_2_O_3_ was used as the model in the XAFS fitting, the fit was far from perfect (Figure 5b). There must be other atoms (Ni or Ce) around the Ni core absorber that affect its scattering. The DFT results of Section 3.5.3.1 also confirm the tendency of Ni to remain as Ni^2+^ in γ-Al_2_O_3_. Among the Ni^2+^-doped structures, those with Ni doped in an octahedral–octahedral site combination have the most negative Δ*E*_formation_. While this suggests that the octahedral–octahedral combination is the more energetically favorable site arrangement (as opposed to tetrahedral–tetrahedral), both XAFS and DFT simulations predict that clustering of Ni (two or more Ni atom doping) is favorable relative to single Ni atom doping. The observed disparity in Ni location in alumina between XAFS fitting and DFT calculations can be attributed to the inherent uncertainties present in both processes and the possible presence of additional Ni clustering or Ce atom doping.

The kinetics results reinforce this interpretation of the active surface structure (Table 1), even for the catalysts prior to their final stabilized forms. In previous work we have shown that for good DRM catalysts two different Δ*E* groupings are evident, high (>104 kJ/mol) and low (<80 kJ/mol).^116^ These ranges are distinguished by the capacity to generate high OV and activate CO_2_ independently of the transition-metal sites. As CeO_2_ is superior in this regard, we expect catalysts with more CeO_2_ mixed into the surface layer to give lower observed Δ*E*′s. This further suggests that the thinner ALD overcoats should exhibit lower Δ*E*′s. This is exactly the behavior observed in Table 1.

Ce XPS (Figure S9) was performed to analyze the electronic properties of Ce near the surface. On going from fresh to used 0.5 nm ALD-CZA40 the XPS-determined Ce^3+^ concentration increased from 35 to 71%, and also showed a marked increase in total surface Ce concentration. This is consistent with CeO_2_, which has been doped with some Ni.^13,20,76,99^ By examining the results of Ni K-edge XANES/XAFS (especially the low Ni–Ni coordination numbers), the Ce XPS results, and the TEM results, it is hypothesized that there was gradual mixing of CeO_*x*_ with the alumina overlayer, that most of the Ni is highly dispersed, some of it is in proximity to this CeO_*x*_ oxide, and this gradual mixing is associated with the significant changes in DRM and RWGS kinetics observed in the first 1–2 days of reactor operation.

The DFT results of Section 3.5.2 (Figures 8–10) also support the thermodynamic favorability of CeO_*x*_ overlayer formation on γ-Al_2_O_3_. Collectively, the DFT doping studies indicate that well-defined conformal ceria layers are not expected to form on the (100) Al_2_O_3_ facet, but can form on the (110) facet, and also that there can be Ce doping into Al_2_O_3_. The DFT results of Section 3.5.2 confirm the enhanced favorability of Ce^4+^ reduction after Ni incorporation, as also observed in the TPR and XPS studies. Furthermore, the results of Table 5 suggest that a single Ni atom incorporation does not enhance the surface reductivity, whereas Ni clustering does. This is in agreement with the TPR results of ALD-coated Ni-CZA (Figure 4), i.e., the observation that Ni incorporation into the ceria lattice during use led to the downward shift of the TPR peak, generating a higher Ce^3+^ content at a lower temperature.

The intimacy of Ce–Al mixing is exemplified by both the STEM images indicating poor crystallization of both Al_2_O_3_ and CeO_2_ in used catalysts, by the lack of an XRD signature for the Al_2_O_3_ overcoats (Figures 3 and S8), and by the TPR results (Figure 4), showing a merger of reduction peaks in the overcoated (especially the 0.5 nm) catalysts. CeO_2_ and Al_2_O_3_ can mix at elevated temperatures, ultimately forming a CeAlO_3_ phase.^117,118^ There are several regions of the TEMs so indistinct that they are consistent with such a disordered mixed-oxide structure, the exact nature of which cannot be discerned from the characterization results.

### 4.3 Comparison of ALD-CZA40 to Other Overcoated or Core–Shell DRM Catalysts

Core–shell architectures are now common in DRM. As previously mentioned, these architectures are designed to suppress deactivation or in some cases increase DRM activity at lower temperatures. While this work is based on the use of ALD to deposit an overlayer of one oxide on top of a mixture of Ni/second oxide, it is worth realizing that there are other ways to do this and that some of these other methods also give core–shell microstructures. Thus, it is important for the results presented here to be analyzed in context with both ALD-deposited and other core–shell DRM catalysts. Table 8 presents DRM turnover frequencies (TOF, defined here as mol CH_4_ converted per second per total mol Ni), the H_2_/CO ratio, and temperature for 0.5 nm ALD-CZA40 along with other core–shell catalysts. Catalysts with a TOF > 0.25 operating in the 700–800 °C range with some proven long-range stability and with H_2_/CO > 0.7 are arbitrarily chosen as the best representative DRM catalysts; there are not many of these in the open literature. However, some other catalysts are included in Table 8 that do not exactly meet these criteria, for comparison purposes.

**Table 8:** Turnover Frequency (Total Ni Atom Basis) and H_2_/CO of Core–Shell-type DRM Catalysts

catalyst (molar basis)	*T* °C	TOF s^–1^	H_2_/CO	ref
0.5 nm ALD-CZA40	750	0.28	0.98	this work
0.015Ni/0.025Mg/0.0062Al/ZrO_*x*_	800	1.94	0.78	(119)
Ni0.018/AlO_*x*_ (ALD)	700	1.0	N/A	(51)
Ni0.018/AlO_*x*_/LaO_*x*_ (ALD)	700	1.0	N/A	(53)
Ni0.014/AlO_*x*_ (ALD)	700	0.51	N/A	(52)
Ni0.038/Ce0.068/SiO_*x*_	700	0.17	0.67	(50)
(core–shell)	750	0.39	0.89	
Ni0.023/SiO_*x*_ (core–shell)	750	0.27	0.87	(120)

It can be seen from Table 8 that the TOF of 0.5 nm ALD-CZA40 is well below that of another Al_2_O_3_ ALD-prepared catalyst (third row of the table). However, this catalyst deactivated very quickly with a 20% loss of activity in 60 h. By comparison, 0.5 nm ALD-CZA40 did not lose activity through 140 h time onstream. The other Al_2_O_3_ ALD-prepared catalyst showed an induction period similar to ours before maximum activity was achieved, and the induction period was attributed to the reduction of NiAl_2_O_4_ to separate phases of Ni^0^ and Al_2_O_3_. However, this is not the case in our work because there is no evidence of bulk NiAl_2_O_4_ in any of our catalysts, fresh or used.

Ahn et al. continued the work on ALD-prepared DRM catalysts by adding La to the Al_2_O_3_ overlayer to fill cation vacancies in γ-Al_2_O_3_.^53^ This was shown to eliminate the induction period seen before. However, this catalyst lost ∼20% of its activity in ∼40 h.

Tathod et al. developed a highly active Ni/MgAl_2_O_4_/Zr catalyst for DRM.^119^ While the activity of this catalyst is far superior to any known Ni-based DRM catalyst, there are some drawbacks. First, it was operated at 800 °C, where it is easier to attain high DRM activity, high H_2_/CO ratio, and minimal coking based on equilibrium considerations. But even at this highly favorable temperature, the sample is far less selective (0.78 H_2_/CO ratio) than the 0.5 nm ALD-CZA40 catalyst. Finally, this Ni/MgAl_2_O_4_/Zr catalyst is less stable than ours.

The Ce-based DRM core–shell catalysts have become popular due to their ability to form an active CeO_*x*_ phase to enhance Ni-site activity, attributed to the formation of OV in CeO_*x*_ (from doped Ni, e.g.). These OVs are known to activate CO_2_. Das et al. synthesized a Ni/SiO_2_@CeO_2_ core–shell material that was active at 700–750 °C.^50^ While its activity at 750 °C was greater than 0.5 nm ALD-CZA40, the latter is 9% (absolute basis) more selective.

Core–shell catalysts of Ni@oxide have been studied, as well. Han et al. coated Ni nanoparticles with a SiO_2_ shell.^120^ This catalyst exhibited approximately the same activity as 0.5 nm ALD-CZA40 (0.28) and showed excellent stability but was again less selective by 12%. For DRM, selectivity and stability are usually considered more important than raw activity, because H_2_ is a more valuable product than CO, and the catalysts would have to operate for long periods between regenerations (or replacement) to make the process economical.

### 4.4 Impact of the Al_2_O_3_ Overcoat

**Figure 13:**
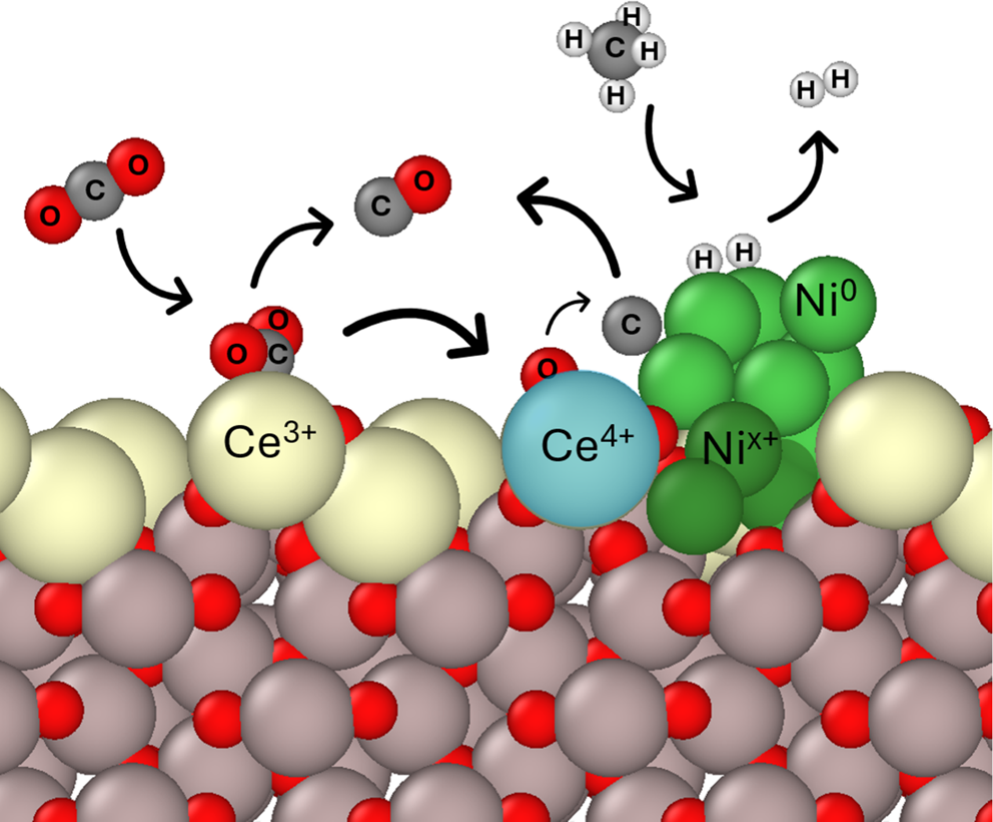
Schematic showing an overview of the surface of the coated catalysts used for DRM.

The deposition of conformal ALD films, especially ultrathin films, is dependent on the reaction affinity of the precursor with available surface ligands. A cartoon of the working catalyst surface is shown in Figure 13. The preferential reaction of TMA with the hydroxy-terminated oxide substrate is believed to leave the prereduced Ni particles uncoated when thin layers (∼<1 nm) are deposited. Thicker ALD layers eventually creep over smaller Ni particles, which can still be exposed to gases during final reduction and DRM. Our DFT results above suggest that the ultrathin Al_2_O_3_ layers then mix with the Ce/Zr substrate when exposed to reaction conditions, forming an amorphous, thin (1–2 ML) CeAlO_*x*_/Al_2_O_3_ surface layer. The thin CeAlO_*x*_ layer serves to inhibit the RWGS reaction by reducing hydrogen spillover, since more Ce is stabilized in the 3+ state. However, oxygen uptake via oxidation of Ce^3+^ through CO_2_ adsorption and transport to the metal-oxide interface are not significantly impacted. This can be seen from the activity of the catalysts and the modest increase in activation energy (Table 1) for thin layers (43 kJ/mol) compared to the thicker layer (86 kJ/mol) compared to reactions over nonreducible substrates (>100 kJ/mol). This dual nature of thin CeAlO_*x*_/Al_2_O_3_ layers is achieved by the generation of thin, miscible coatings on reducible substrates.

## 5 Conclusions

An ALD-deposited Ni-containing catalyst, made by overcoating Al_2_O_3_ on Ni/CeO_2_–ZrO_2_, compares well to other core–shell and ALD-prepared catalysts for dry reforming by all significant metrics, in particular, giving an H_2_/CO ratio near one. The mixture of a nonreducible and reducible support in a core–shell architecture, with Ni initially sandwiched between them but diffusing to the surface during both reduction and DRM, is a promising path to achieving highly active, selective, and stable DRM catalysts.

An optimal Al_2_O_3_ overlayer thickness of ∼0.5 nm was determined. The ALD overlayer increased the lifetime of the uncoated catalyst by at least 10-fold. The coking rate was minimized, and the Ni clusters of the used catalyst were smaller and less reducible. This structure had the added benefit of decreasing the RWGS rate and increasing the DRM rate. We speculate that the decrease in the RWGS rate reflects reduced H_2_ spillover to the coated, Al_2_O_3_-containing, surface.

The induction time seen during reactor testing at high conversion is associated with the migration of CeO_*x*_ from bulk CZA to the surface, generating a mixed CeO_2_–Al_2_O_3_ phase. It is likely that this mixed oxide is where the Ni^2+^ and small Ni clusters, which are a significant fraction of the total Ni, are located. While this active Ni would prefer association with pure Al_2_O_3_ rather than pure CeO_2_, the mixing of the two oxides at the surface renders the actual surface more complex.

## Supporting Information Available

Reduction profiles; reactor and analytical data; DFT volume optimization of bulk structures; XRD and morphological data; effects of GHSV on reactions; TPO, TPR, and Ce 3d XPS results; Ni K-edge XANES; Ni K-edge XAFS fits; additional TEM data; details of DFT on Ce doping into Al_2_O_3_, and Ni doping into Al_2_O_3_ and CeO_2_ (PDF)
