# Supplementary material for: Improved Selectivity and Stability in Methane Dry Reforming by Atomic Layer Deposition on Ni-CeO2–ZrO2/Al2O3 Catalysts
Source: ACS Catal. 2024 May 30;14(12):9115–33. doi: 10.1021/acscatal.4c02019 (PMC11197040; doi:10.1021/acscatal.4c02019)
Supplement: Supplementary file 1 — cs4c02019_si_001.pdf [file cs4c02019_si_001.pdf]

Supporting Information

Improved Selectivity and Stability in Methane Dry Reforming by Atomic Layer Deposition onto Ni-CeO_2-ZrO_2/Al_2O_3 Catalysts

Jonathan Lucas[1], Nirenjan Shenoy Padmanabha Naveen[2], Michael J. Janik[2], Konstantinos Alexopoulos[2], Gina Noh[2], Divakar Aireddy[1], Kunlun Ding[1], James A. Dorman[1],* and Kerry M. Dooley[1*]

1. Department of Chemical Engineering, Louisiana State University, Baton Rouge, Louisiana 70803, United States
2. Department of Chemical Engineering, The Pennsylvania State University, University Park, PA 16802, United States
**Corresponding Author: Dooley@LSU.edu, jadorman@gmail.com*

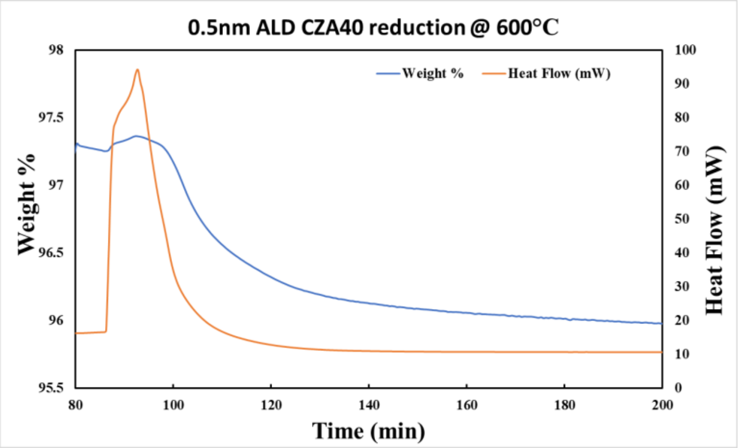

Figure S1. *Reduction profile of 0.5nm ALD CZA40, 5% H_2/N_2, prior to DRM screening in DSC/TGA. Final curves of wt% and heat flow show essentially complete reduction.*

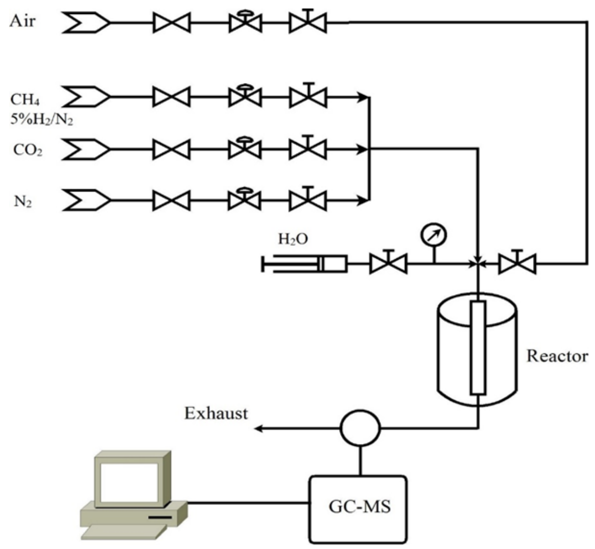

Figure S2. *Schematic of the reactor system for DRM.*[2] *GC parameters in Table S1. Reprinted from ref 2 with permission from author Changyi Jiang.*

Analytical (GC) details

The gas samples from the reactor were analyzed by an Agilent 6890N GC/MS. Three 1/8" molecular sieve packed columns, a Wasson K1 (6 ft), K2 (7 ft), and K2S (2 ft) in series were used to separate hydrogen from the rest of the gaseous mixture, with the H_2 analyzed by a thermal conductivity detector. A 1 mL sample was injected using N_2 as the carrier gas at 30 psig. Another 1 mL sample was injected into a 0.53 mm, 50 m capillary column (Wasson KC080) to separate N_2, CO, CO_2, CH_4 and water, also analyzed by a thermal conductivity detector. Helium was the carrier gas, controlled by electronic flow controller. A third sample was injected (as needed) to a 100 m, 0.25 mm Wasson KC066 column which separated all components and interfaced to the MSD, which was used for confirmation component identities. Helium was the carrier gas, controlled by electronic flow controller. The oven program and TCD details are provided below. GC calibration factors and retention times are presented in Table S1.

Front Inlet – Split; 120°C; P: 4.5 psi; column flow 0.5 mL/min; total flow: 7.8 mL/min; split ratio = 10:1; split flow = 4.8 mL/min

Back Inlet – Split; 120°C; P: 7.4 psi; column flow 5.4 mL/min; total flow: 79 mL/min; split ratio = 13.1:1; split flow=70.4 mL/min 78

Front Detector – TCD; 200°C; 16.5 mL/min ref. flow; 11 mL/min makeup flow; Gas = He

Back Detector – TCD; 200°C; 20 mL/min ref. flow; 3 mL/min makeup flow; Gas = N2; Negative polarity

GC Oven Program – Initial Temp = 30 °C; Initial time = 7 min; Rate1 = 10 °C/min to 100 °C; 5 min hold; Rate2 = 10 °C/min to 130 °C; 16 min hold; Post Temp = 30 °C;

Post Time = 15 min; Total run time = 38 min

Packed Column Auxiliary Oven Temperature Program – Initial Temp = 50 °C; Initial time = 5 min; Rate1 = 10 °C/min to 120 °C, hold 11 min; Rate2 = 10 °C/min to 50°C; Chase heater – 100 °C; Injector (GSVs) oven – 200 °C

Table S1. GC Calibration Factors and Rentention Times

Component	GC Factor Mol (μmol)/area (MM)	Rentention Time (min)
CO	0.46	3.6
CO_2	0.44	8.9
N_2	0.37	3.5
CH_4	0.69	4.3
H_2O	0.55	21.3
H_2	0.082	2.8[1]

[1]This is the retention time for the packed columns.

Rate/Conversion Calculations from TGA/DSC Data

Aspen HYSIS® allows computation of the heat of reaction at a given conversion. The % conversion in DRM was varied until the computed heat matched the measured heat from the DSC. We simulated the combined DRM/RWGS reactions in HYSIS as well. The heat flow for DRM is 7.2-7.5 times that of RWGS (over the range 650-800 °C). Therefore since the RWGS reaction is at least three times slower than the DRM rate (based on observed H_2/CO ratios), the error in the computed DRM conversion by neglecting the RWGS in the calculation is < 5%. The conversions

so obtained were all in the differential range (< 5%), which allows for direct computation of the rate assuming a differential reactor.

Volume optimization of bulk structures

All the bulk structures (pure and metal-doped oxides) were optimized for both ion positions and unit cell volume. The volume of the initially converged bulk structure was varied by increasing/decreasing the scaling factor of the unit cell across an appropriate range with a step change of 0.005, and structural optimization of the scaled structure was performed for every chosen scaling factor. The 'DFT Energy (eV) vs scaling factor' plot was utilized to find the scaling factor giving the lowest DFT energy. The Birch-Murnaghan equation was used to fit the experimental data to a parabolic function. An example plot for the data fitting carried out for the bulk NiO structure is shown in **Figure S3**.

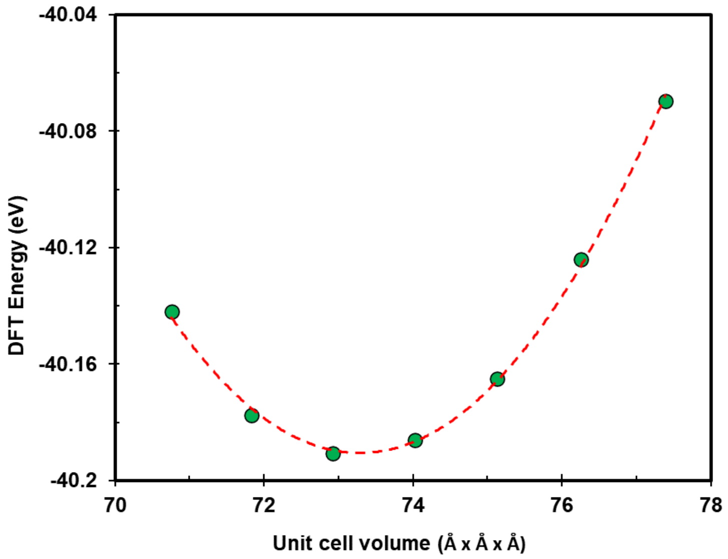

Figure S3. *'DFT Energy vs volume' curve for the bulk NiO structure*

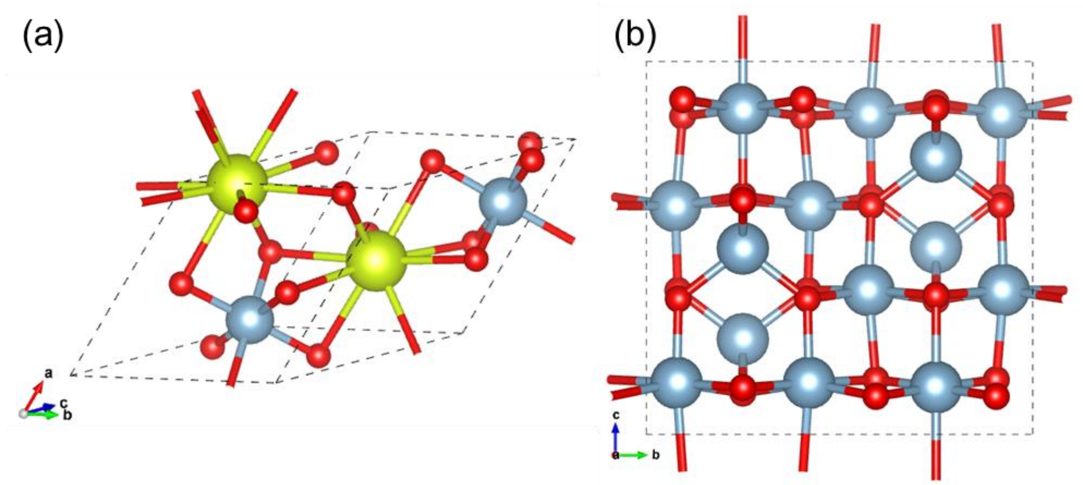

Figure S4. *Unit cells for bulk* ***(a)*** *cerium aluminate ($CeAlO_3$) and* ***(b)*** *γ-Al_2O_3. The yellow, light blue, and red spheres represent Ce, Al, and O atoms, respectively.*

Table S2. Structural details and k-point grids of bulk models.

**Bulk type**	**Structural details**	**Dimensions,** Å x Å x Å	**k-point grid**
γ-Al_2O_3	Space group: P1<br>Unit cell: $Al_{16}O_{24}$<br>$\alpha = \gamma = 90^o$; $\beta = 90.59^o$	5.58 x 8.40 x 8.07	6 x 3 x 3
Ce_2O_3	Space group: P-3m1<br>Unit cell: Ce_2O_3<br>$\alpha = \beta = 90^o$; $\gamma = 120^o$	3.93 x 3.93 x 6.09	4 x 4 x 2
CeO_2	Space group: Fm-3m<br>Unit cell: Ce_2O_4<br>$\alpha = \beta = \gamma = 90^o$	5.52 x 5.52 x 5.52	2 x 2 x 2
Ni	Space group: Fm-3m<br>Unit cell: Ni_4<br>$\alpha = \beta = \gamma = 90^o$	3.58 x 3.58 x 3.58	3 x 3 x 3
NiO	Space group: Fm-3m<br>Unit cell: Ni_4O_4	4.18 x 4.18 x 4.18	2 x 2 x 2

	α = β = γ = 90°		
$CeAlO_3$	Space group: R-3c<br>Unit cell: $Ce_2Al_2O_6$<br>α = β = γ = 60.05°	5.35 x 5.35 x 5.35	2 x 2 x 2

Table S3. Structural details and k-point grids of slab models.

**Surface**	**Slab details**	**Surface dimensions, Å x Å**	**k-point grid**
$\gamma - Al_2O_3(100)$	2 layers deep	5.58 x 8.40	6 x 3 x 1
$\gamma - Al_2O_3(110)$	2 layers deep	8.07 x 8.40	3 x 3 x 1
$CeO_2(111)$	2 layers deep	7.73 x 2.83	2 x 2 x 1

Table S4. N_2 physisorption BET results and XRD calculated crystallite sizes

Sample	State	Crystallite Size[1] (nm)	Surface Area (m^2/g)	Pore Volume (cm^3/g)	Pore Width (nm)
CZA40	Fresh	5.7	95	0.6	31
	Used	6.6	--	--	--
0.3nm ALD-CZA40	Fresh	6.8	71	0.51	27
	Used	--	167	0.36	--
0.5nm ALD-CZA40	Fresh	6.7	72	0.63	27
	Used	6.4	77	0.54	26
1.2nm ALD-CZA40	Fresh	6.5	61	0.42	27

[1]Crystallite sizes calculated using the Scherrer equation, using Ce (111)

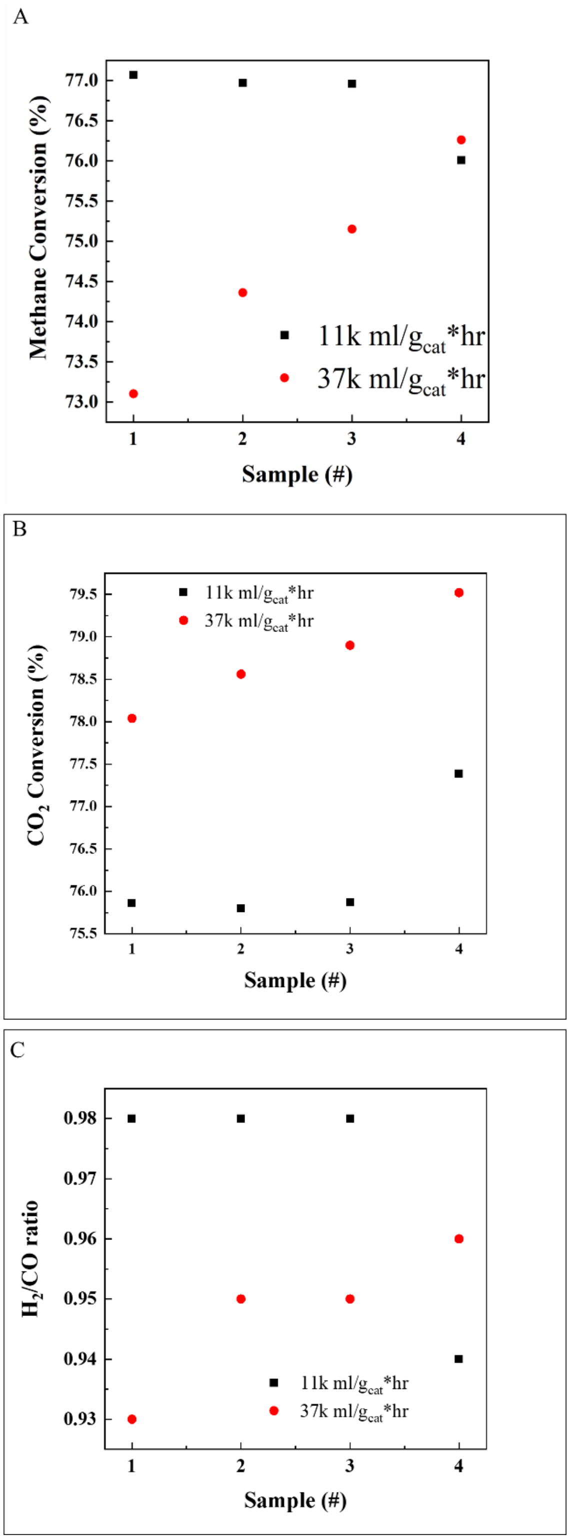

Figure S5. *Effect of increasing GHSV on: a.) CH_4 conversion; b.) CO_2 conversion; c.) H_2/CO ratio of 0.5nm ALD-CZA40*

Temperature Programmed Oxidation (TPO) coke analysis of used catalysts

TPO coke analysis was done using a TA SDT Q600 Differential Scanning calorimetry (DSC)/Thermogravimetric Analyzer (TGA). Used catalysts were temperature ramped from 100 to 650 °C at 10 °C/min under 75 mL/min of air flow. Catalysts were then held at 650 °C for 50 min. Coking rates were calculated using the % weight change.

Temperature Programmed Reduction (TPR) of ALD Catalysts

TPR was done using a ChemBET Pulsar TPR/TPD. Catalysts were brought to 100 °C at 10 °C/min prior to TPR. Then they were ramped at 10 °C/min to 900 °C under 40 mL/min of 5% H_2/N_2, and held at 900 °C for 15 min.

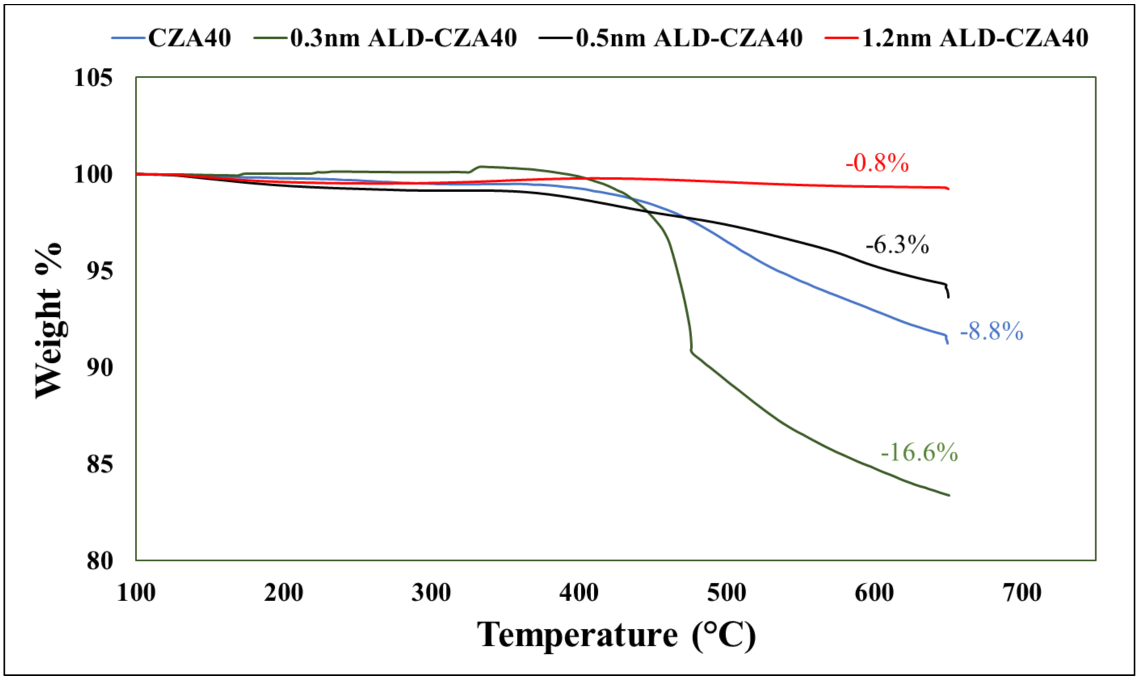

Figure S6. *TPO coke analysis of used ALD catalysts. Samples were ramped from 100 to 650 °C with a 10 °C/min ramp rate at 75 mL/min air flow.*

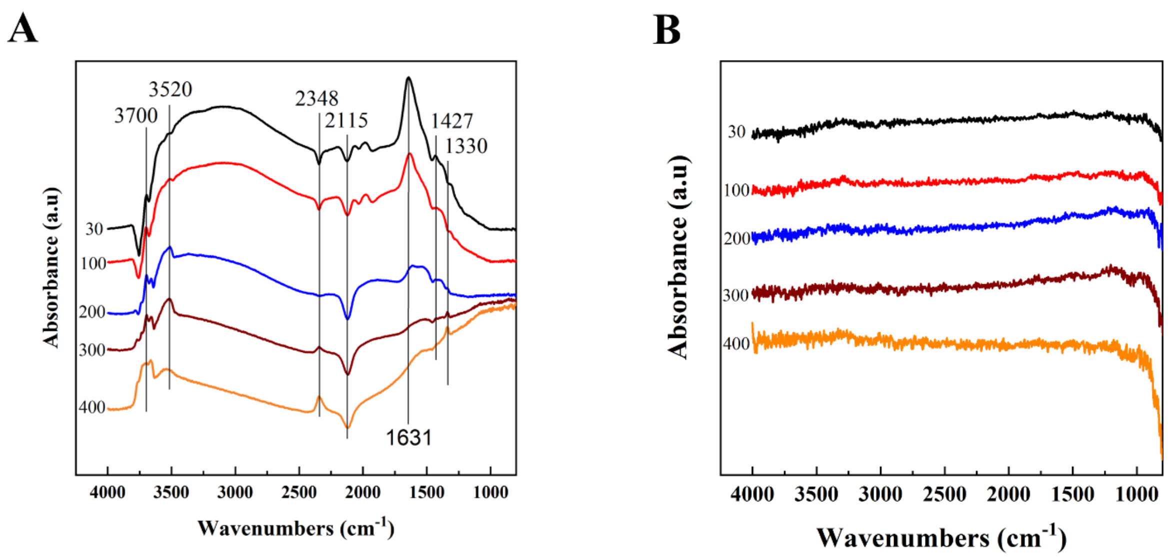

Figure S7. *A) CO_2 DRIFTS of uncoated CZA40 B.) CO_2 DRIFTS of 0.5nm ALD-CZA40. DRIFTS collected at temperatures ranging from 30-400 °C after adsorption at 30 °C.*

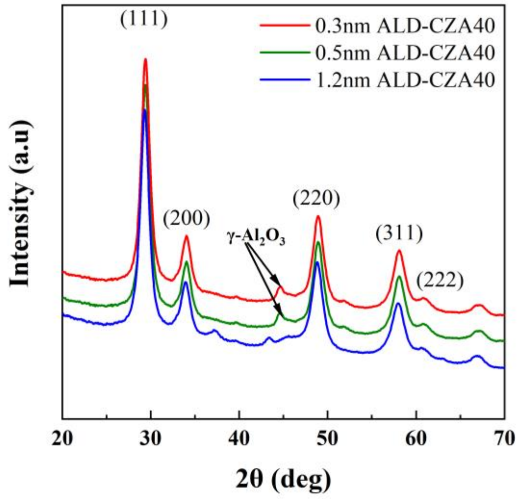

Figure S8. *XRD analysis of fresh 0.3nm (red line), 0.5nm (green line), 1.2nm (blue line) ALD-CZA40. The numeric labels refer to planes of the fluorite lattice.*

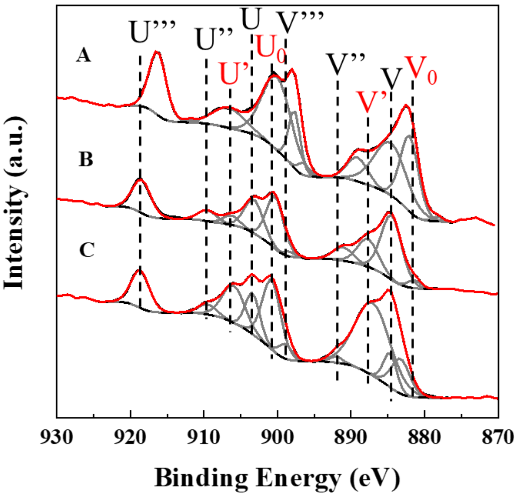

Figure S9. *Ce 3d XPS spectrum of* ***(a)*** *CZA40,* ***(b)*** *fresh 0.5nm ALD-CZA40, and* ***(c)*** *used 0.5nm ALD-CZA40*

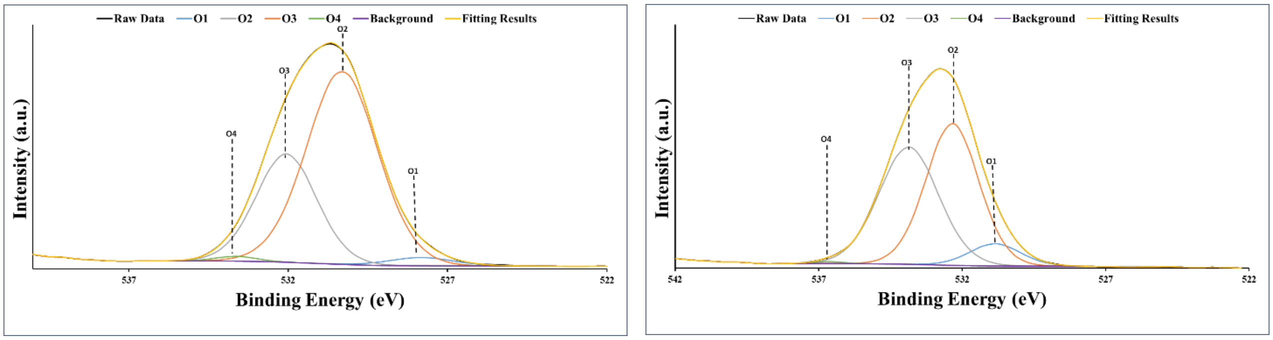

Figure S10. *O 1s XPS spectra for fresh (L) and used (R) 0.5nm ALD-CZA40*

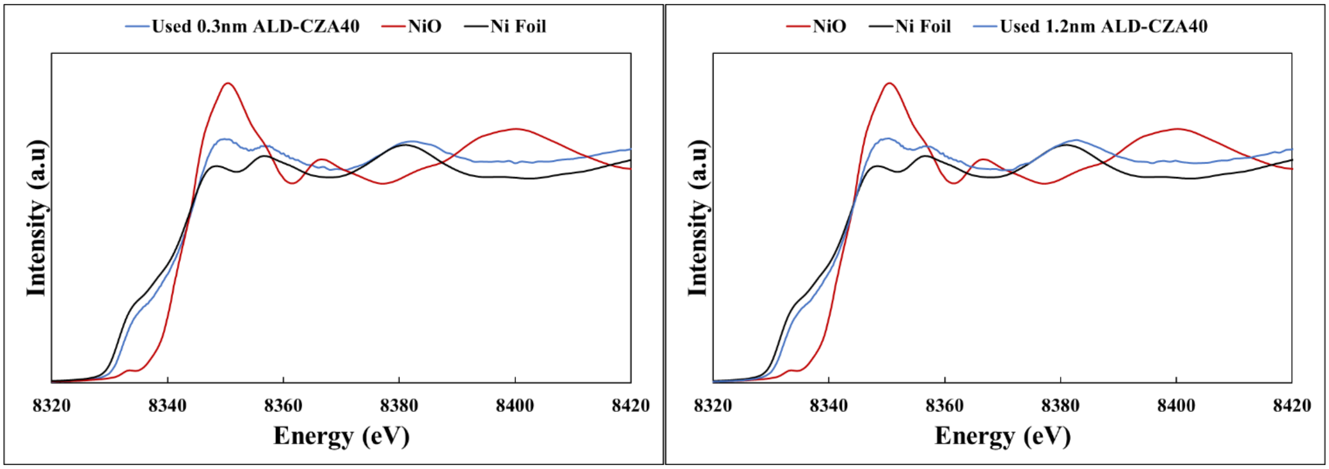

Figure S11. *Ni K-edge XANES for a.) used 0.3nm ALD-CZA40 b.) used 1.2nm ALD-CZA40*

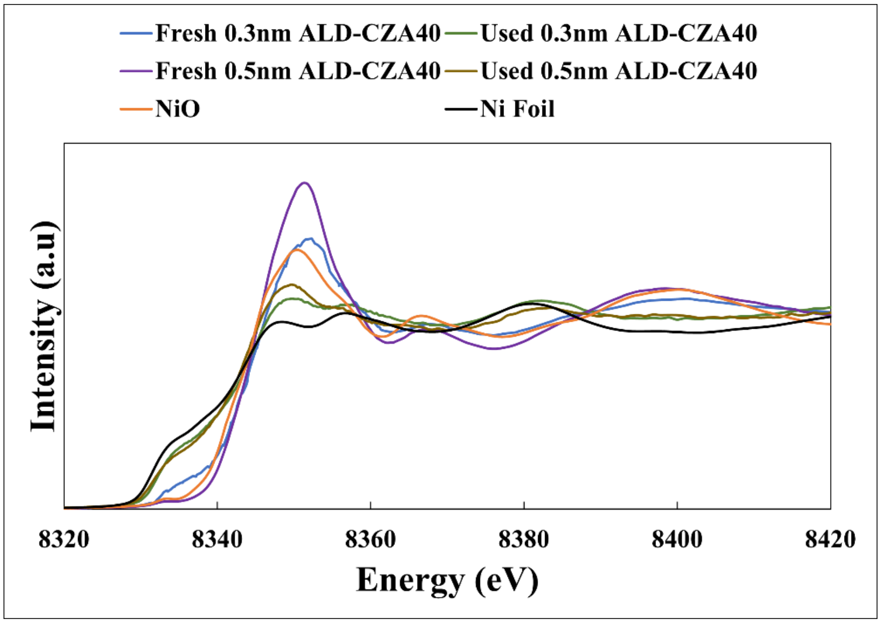

Figure S12. Ni K-edge XANES of fresh/used 0.3nm and 0.5nm ALD-CZA40

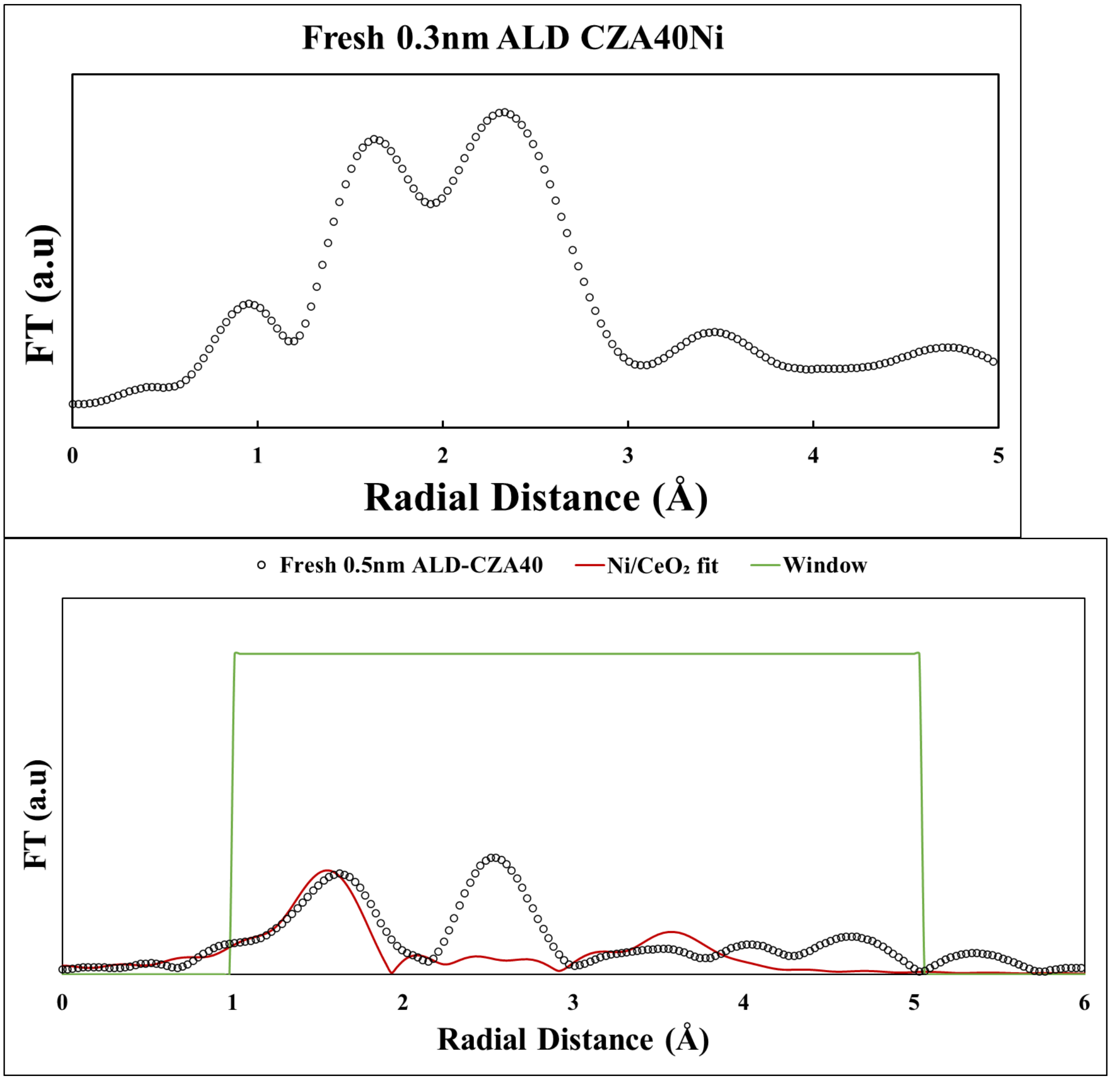

Figure S13. *Top: Ni K-edge XAFS of fresh 0.3nm ALD CZA40; bottom: Ni K-edge XAFS for fresh 0.5nm ALD-CZA40 using a simulated Ni/CeO_2 fit*

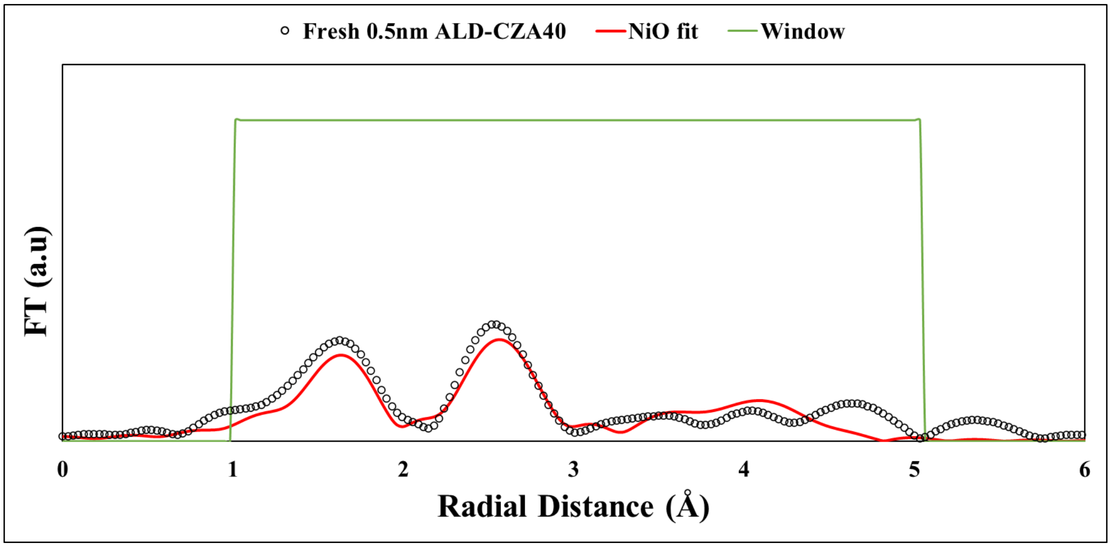

Figure S14. *Ni K-edge XAFS for fresh 0.5nm ALD-CZA40 using a simulated NiO fit*

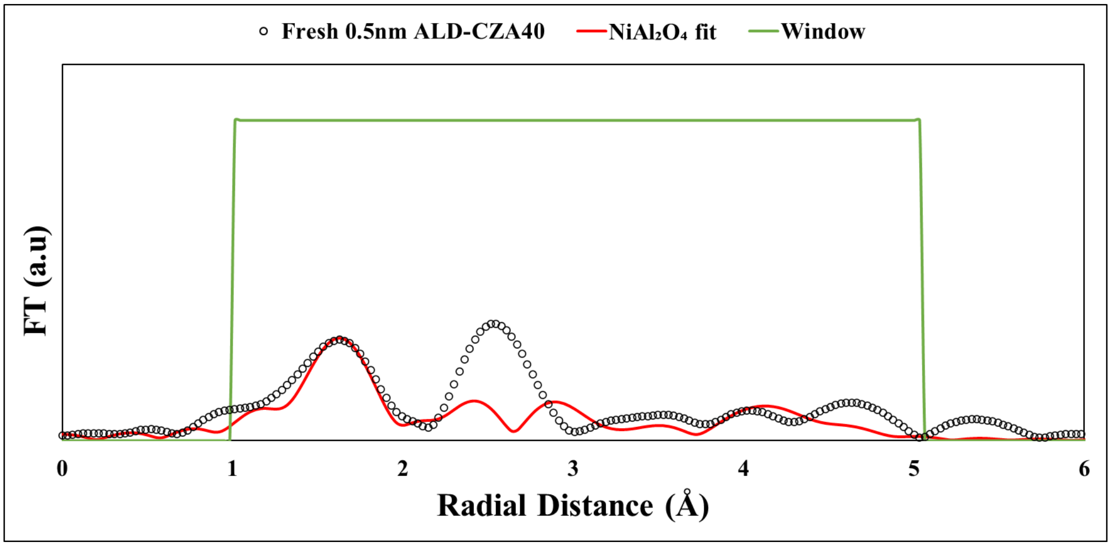

Figure S15. *Ni K-edge XAFS for fresh 0.5nm ALD-CZA40 using a simulated* $NiAl_2O_4$ *fit*

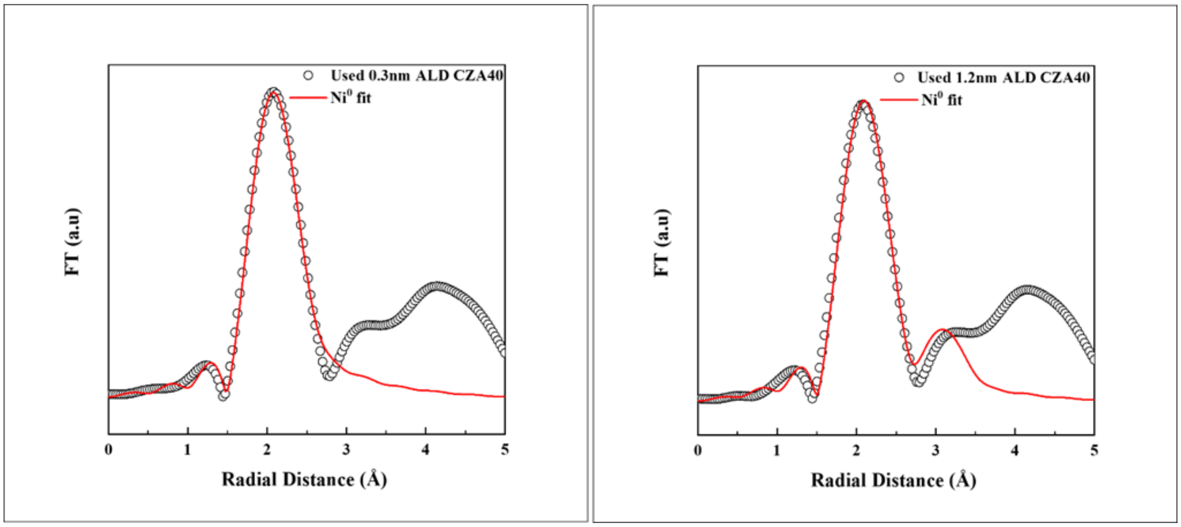

Figure S16. *Ni K-edge XAFS for a.) used 0.3nm ALD-CZA40 b.) used 1.2nm ALD-CZA40. First shell fits only, 1-3 Å.*

Table S5. XAFS fitting results for fresh and used catalysts.[1]

**Sample**	Shell	N	σ^2 ($\AA^2$)	R ($\AA$)	R-factor
Used 0.3nm ALD CZA40	1st Ni-Ni	9.83 ± 0.52	0.00545 ± 0.0014	2.48 ± 0.009	0.013
Used 1.2nm ALD CZA40	1st Ni-Ni	10.12 ± 1.46	0.00528 ± 0.0013	2.44± 0.009	0.015
	2nd Ni-Ni	4.06 ± 1.52	0.00528	3.45	
Fresh 0.5nm ALD CZA40 (NiO fit)	1st Ni-O	6	0.00572 ± 0.0017	2.07 ± 0.013	0.143
	1st Ni-Ni	12	0.01257	2.94 ± 0.014	
	2nd Ni-O	8	0.00572	3.60 ± 0.013	
	2nd Ni-Ni	6	0.01257	4.20 ± 0.014	
	3rd Ni-O	24	0.00572	4.64 ± 0.013	
Fresh 0.5nm ALD CZA40 (Ni/CeO_2 fit)	1st Ni-O	6	0.00630 ± 0.0023	2.00 ± 0.031	0.393
	1st Ni-Ce	12	0.01200 ± 0.0050	3.65 ± 0.042	
	2nd Ni-O	24	0.00630 ± 0.0023	4.48 ± 0.031	
Fresh 0.5nm ALD CZA40 ($NiAl_2O_4$ fit)	1st Ni-O	6	0.00379 ± 0.0021	2.06 ± 0.023	0.368
	1st Ni-Ni	3	0.00829 ± 0.0052	2.86 ± 0.037	
	1st Ni-Al	3	0.00829 ± 0.0052	2.86 ± 0.037	
	2nd Ni-O	2	0.00379 ± 0.0021	3.49 ± 0.023	
	3rd Ni-O	6	0.00379 ± 0.0021	3.62 ± 0.023	
	4th Ni-O	12	0.00379 ± 0.0021	4.58 ± 0.023	
	5th Ni-O	12	0.00379 ± 0.0021	4.67 ± 0.023	
	2nd Ni-Ni	5	0.00829 ± 0.0052	4.95 ± 0.037	
	2nd Ni-Al	7	0.00829 ± 0.0052	4.95 ± 0.037	

[1]For the used catalysts, the fit is to Ni(0) only. N is the coordination number, σ^2 the Debye–Waller factor, R the Ni–O or Ni–Ni distance. The uncertainty in the structural parameters for the first shell obtained from XAFS fitting are given for N, σ^2 and R.

Table S6. XAFS fitting results for fresh and used 0.5nm ALD-CZA40.[1]

**Sample**	Shell	N	σ^2(Å^2)	R (Å)	R-factor
Fresh 0.5nm ALD-CZA40 (one Ni)	1st Ni-O	4	0.00247 +/- 0.003	2.01 +/- 0.04	0.439
	1st Ni-Al	12	0.00453 +/- 0.003	3.15 +/- 0.04	
	2nd Ni-O	12	0.00247 +/- 0.003	3.50 +/- 0.04	
	1st Ni-Ni	4	0.00453 +/- 0.003	3.30 +/- 0.04	
	2nd Ni-Al	12	0.00247 +/- 0.003	4.46 +/- 0.04	
Fresh 0.5nm ALD-CZA40 (two Ni)	1st Ni-O	4	0.00149 +/- 0.002	1.99 +/- 0.03	0.199
	1st Ni-Al	12	0.00652 +/- 0.002	3.16 +/- 0.03	
	2nd Ni-O	12	0.00149 +/- 0.002	3.49 +/- 0.03	
	1st Ni-Ni	4	0.00652 +/- 0.002	3.31 +/- 0.03	
	2nd Ni-Al	12	0.00652 +/- 0.002	3.31 +/- 0.03	
Used 0.5nm ALD-CZA40	1st Ni-O	6	0.0124 +/- 0.00775	2.09 +/- 0.037	0.013
	1st Ni-Ni	8.30 +/- 0.439	0.0006 +/- 0.00154	2.44 +/- 0.009	

[1]N is the coordination number. σ^2 is the Debye–Waller factor, R is the Ni–O or Ni–Ni distance. The uncertainty in the structural parameters for the first shell obtained from XAFS fitting are given for N, σ^2 and R.

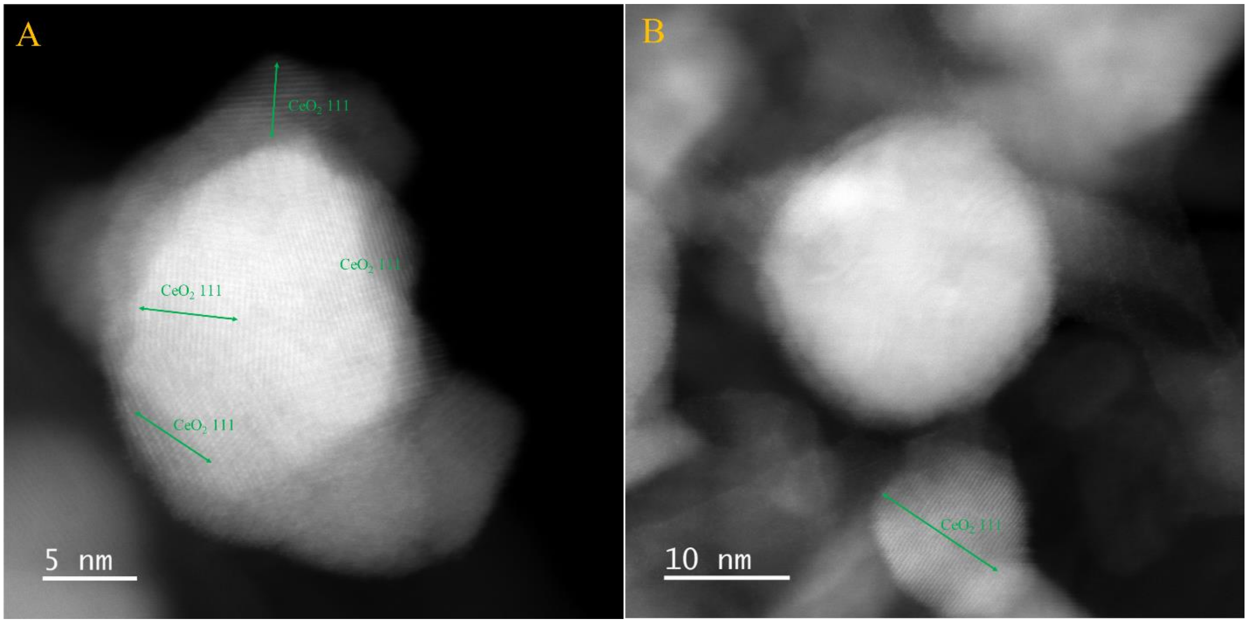

Figure S17. *DF STEM of two regions of used 0.5nm ALD-CZA40*

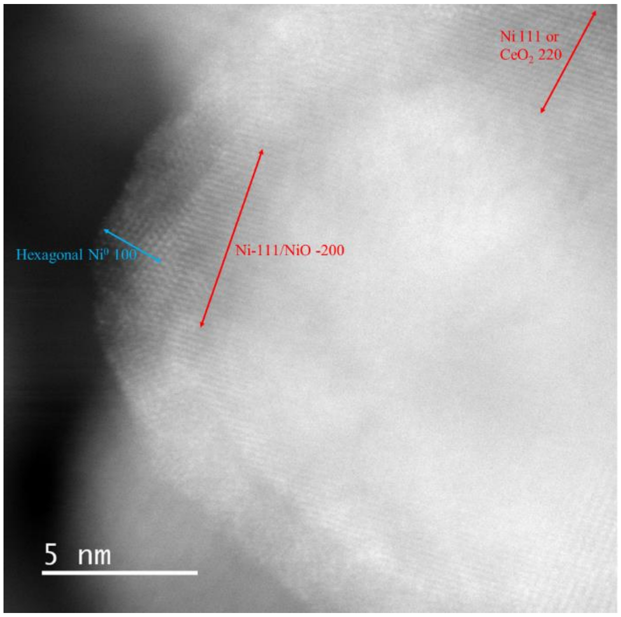

Figure S18. *DF STEM of used 0.5nm ALD-CZA40. A magnified region of Fig. 6b better showing some hexagonal Ni^0.*

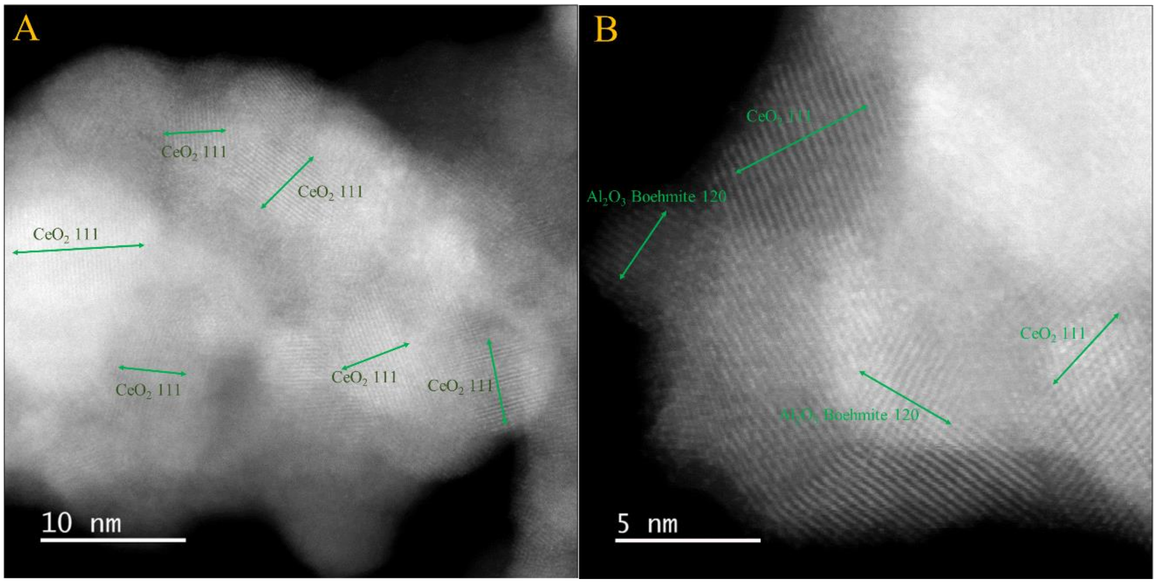

Figure S19. *DF STEM of two regions of fresh 0.5nm ALD-CZA40*

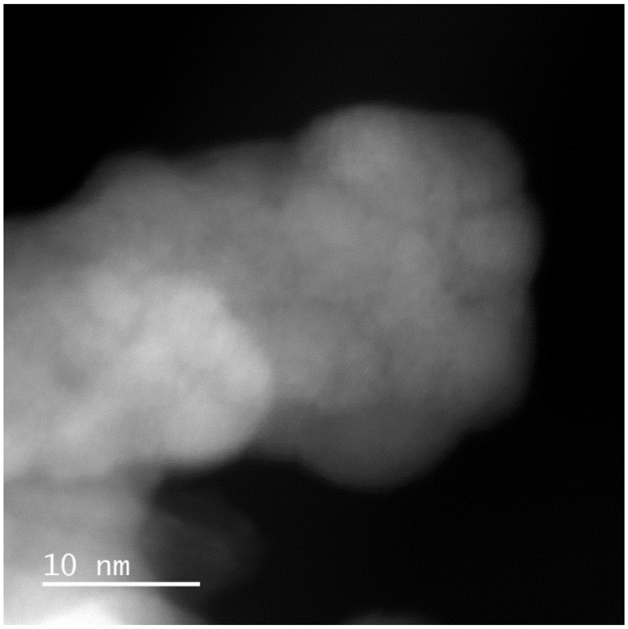

Figure S20. *DF STEM of used CZA40*

Doping sites on (100) and (110) γ-Al_2O_3: The 100 facet has four chemically distinct Al sites, where site 11 (tetrahedrally coordinated) is a near-surface site and the rest of the sites 08, 12 and 13 (penta-coordinated) are present on the surface.

The 110 facet has five chemically distinct Al sites. Site 02 (octahedrally coordinated) and 16 (tetrahedrally coordinated) are situated on a sub-surface level and the rest of the sites, 01, 09 (tetrahedrally coordinated) and 14 (tri-coordinated) are present on the surface.

Ce^{4+} doping on (100) and (110) γ-Al_2O_3: Ce^{4+} doping was considered on (100) and (110) γ-Al_2O_3. Single Ce^{4+} doping was accompanied by adsorption of a hydroxyl group on the surface-doped Ce atom. In multiple Ce substitutions, an O was bridged between 2 surface-doped Ce atoms (even number of substitutions) along with the adsorption of a hydroxyl group (odd number of substitutions).

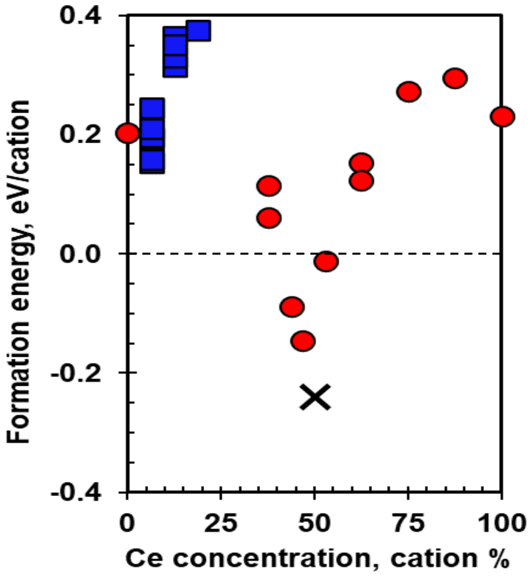

Figure S21. *Formation energy as a function of Ce content for Ce-doped γ-Al_2O_3 (blue squares), $CeAlO_3$ (black X), and Ce- or Al-doped $CeAlO_3$ (red circles).*

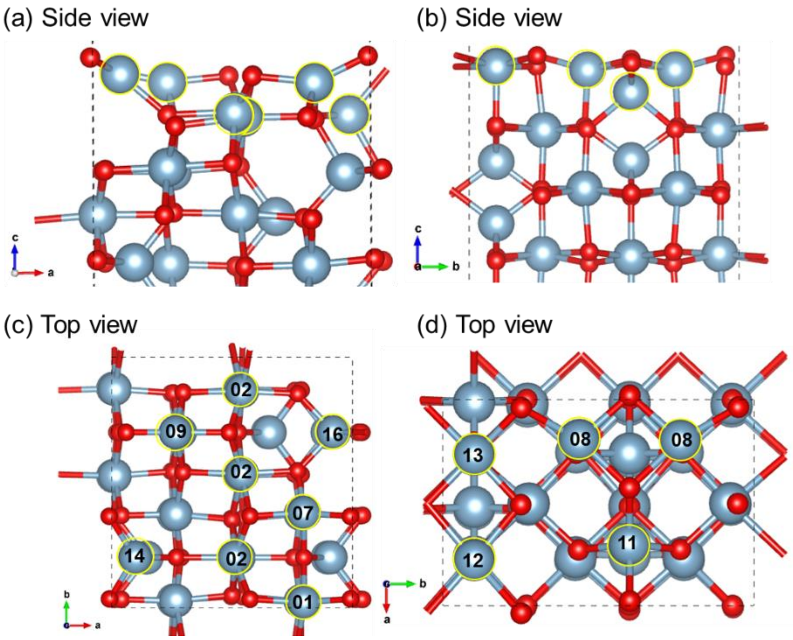

Figure S22. ***(a)*** *&* ***(b)*** *side view of the (110) and (100) stoichiometric facets of γ-Al_2O_3 (only the top half of the slab is shown),* ***(c) & (d)*** *surface Al sites (top view) on the 110 and 100 facets of γ-Al_2O_3, the numbers indicate the surface Al sites*

Table S7. Reaction equations for Ce^{3+}-doped γ-Al_2O_3 surfaces

1 Ce (3.13%) doping	$\mathbf{Al_{32}O_{48\,(surface)} + \frac{1}{2}Ce_2O_{3\,(bulk)} \rightarrow Al_{31}Ce\,O_{48\,(surface)} + \frac{1}{2}Al_2O_{3\,(bulk)}}$	**(S1)**
2 Ce (6.25%) doping	$Al_{32}O_{48\,(surface)} + Ce_2O_{3\,(bulk)} \rightarrow Al_{30}Ce_2\,O_{48\,(surface)} + Al_2O_{3\,(bulk)}$	**(S2)**
3 Ce (9.38%) doping	$Al_{32}O_{48\,(surface)} + \frac{3}{2}Ce_2O_{3\,(bulk)} \rightarrow Al_{29}Ce_3O_{48\,(surface)} + \frac{3}{2}Al_2O_{3\,(bulk)}$	**(S3)**
4 Ce (12.5%) doping	$Al_{32}O_{48\,(surface)} + 2Ce_2O_{3\,(bulk)} \rightarrow Al_{28}Ce_4\,O_{48\,(surface)} + 2Al_2O_{3\,(bulk)}$	**(S4)**

Table S8. Reaction equations for Ce^{4+}-doped γ-Al_2O_3 surfaces

1 Ce (3.13%) doping	$\mathbf{Al_{32}O_{48}}_{\mathbf{(surface)}} + \frac{\mathbf{1}}{\mathbf{2}}\mathbf{Ce_2O_3}_{\mathbf{(bulk)}} + \mathbf{H_2O_{(g)}} \rightarrow \mathbf{Al_{31}Ce\,O_{49}H}_{\mathbf{(surface)}} + \frac{\mathbf{1}}{\mathbf{2}}\mathbf{Al_2O_3}_{\mathbf{(bulk)}} + \frac{\mathbf{1}}{\mathbf{2}}\mathbf{H_2}_{\mathbf{(g)}}$	**(S5)**
2 Ce (6.25%) doping	$Al_{32}O_{48}{}_{(surface)} + Ce_2O_3{}_{(bulk)} + H_2O_{(g)} \rightarrow Al_{30}Ce_2\,O_{49}{}_{(surface)} + Al_2O_3{}_{(bulk)} + H_2{}_{(g)}$	**(S6)**
3 Ce (9.38%) doping	$Al_{32}O_{48}{}_{(surface)} + \frac{3}{2}Ce_2O_3{}_{(bulk)} + 2H_2O_{(g)} \rightarrow Al_{29}Ce_3O_{49}H_{(surface)} + \frac{3}{2}Al_2O_3{}_{(bulk)} + \frac{3}{2}H_2{}_{(g)}$	**(S7)**
4 Ce (12.5%) doping	$Al_{32}O_{48}{}_{(surface)} + 2Ce_2O_3{}_{(bulk)}2H_2O_{(g)} \rightarrow Al_{28}Ce_4\,O_{50}{}_{(surface)} + 2Al_2O_3{}_{(bulk)} + 2H_2{}_{(g)}$	**(S8)**

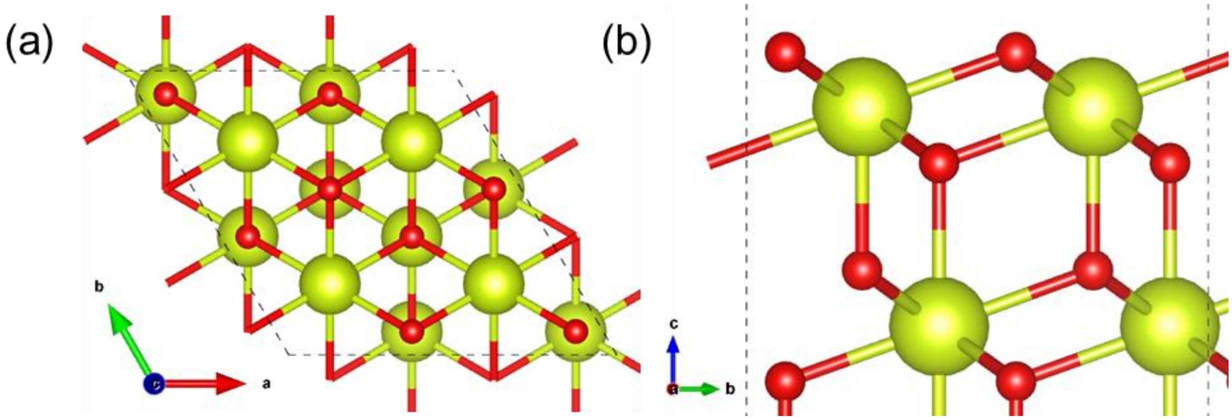

Figure S23. *The (111) CeO_2 facet (a) top view, (b) side view (only the top two atomic layers are shown)*

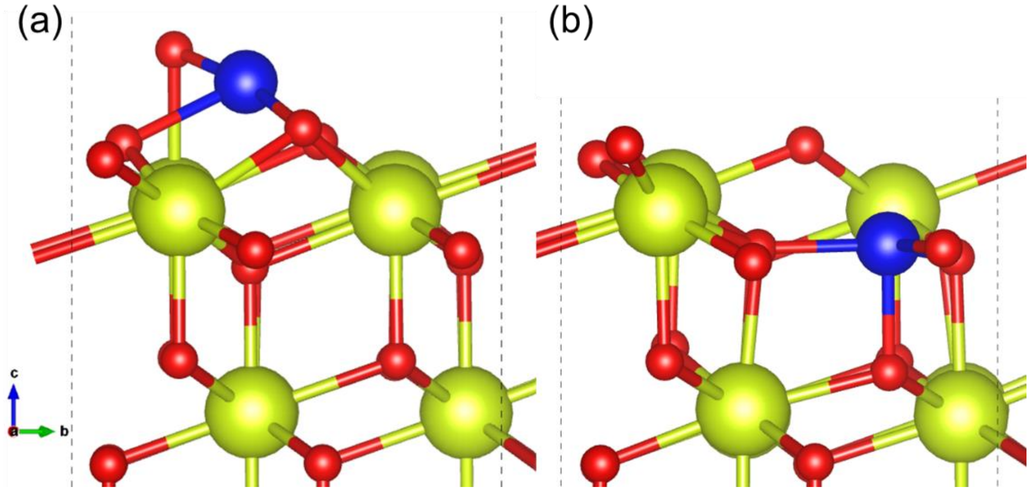

Figure S24. *(a) NiO adsorbed (111) CeO_2 surface (side view) (b) 1 Ni-doped (111) CeO_2 surface with 1 oxygen vacancy (side view); Ni atoms are represented by dark blue spheres, remaining legends defined previously*

Ni-doped and Ni-adsorbed ceria surfaces: All Ce atoms on the surface are crystallographically equivalent, thus, the choice of any Ce atom as a doping site should result in the same energies. However, there are three distinct high-symmetry adsorption sites for the (111) facet – the atop Ce site, the atop surface O site, and the atop sub-surface O site. Any other starting configuration will converge to one of these three structures. The atop sub-surface O site was the most stable adsorption site.

For energy comparisons between the doped and adsorbed configurations, we chose NiO adsorbed on the atop sub-surface O site structure as the adsorbed reference structure, and the Ni-doped ceria surface with one oxygen vacancy (created by removing a Ni bonded O) as the doped reference structure. In the doped structure, an oxygen vacancy was considered by removing either an O that is bonded to the doped Ni or from a faraway Ce atom. The most negative doping energy was obtained when the immediately bonded O was removed.

1. Jiang, C.; Akkullu, M. R.; Li, B.; Davila, J. C.; Janik, M. J.; Dooley, K. M., Rapid screening of ternary rare-earth – Transition metal catalysts for dry reforming of methane and characterization of final structures. *Journal of Catalysis* **2019,** *377*, 332-342.

2. Jiang, C; Transition Metal-doped Rare-earth Oxysulfide Catalysts for High Temperature Dry Reforming of Methane, in, Louisiana St. Univ., Louisiana St. Univ., 2020
